# How strongly do moral character inferences predict forecasts of the future? Testing the moderating roles of transgressor age, implicit personality theories, and belief in karma

**DOI:** 10.1371/journal.pone.0244144

**Published:** 2020-12-21

**Authors:** Cindel J. M. White, Ara Norenzayan, Mark Schaller

**Affiliations:** Department of Psychology, The University of British Columbia, Vancouver, British Columbia, Canada; Ghent University, BELGIUM

## Abstract

Three studies (total *N* = 1486) investigated how inferences about a person’s current moral character guide forecasts about that person’s future moral character and future misfortunes, and tested several plausible moderating variables. Inferences about current moral character related (very strongly) to forecasts about future moral character and also (less strongly) to forecasts about future misfortunes. These relationships were moderated by two variables: Relations between inferences and forecasts were somewhat weaker when perceivers made judgments about children, compared to judgments about adults, and relations between character inferences and forecasts about misfortunes were somewhat stronger among perceivers who more strongly believed in karma. In contrast, results provided no evidence of any moderating effects due to perceivers’ beliefs about the stability of moral dispositions (i.e., implicit personality theories). These results show how dispositional inferences, moral judgments, and beliefs about karmic justice interact to shape forecasts about the future.

## Introduction

Human beings are moral judges. We judge other individuals’ actions to be good or bad and on the basis of those actions we also make inferences about those individuals’ moral character—whether they are good or bad people. Human beings are also moral forecasters. On the basis of our inferences about a person’s moral character, we may forecast their future—predicting whether an individual will continue be good or bad in coming years, and also predicting the fortunes and misfortunes that they might experience over the course of their lives. Just how strongly do inferences about someone’s current moral character guide forecasts about their future character and future fortunes? The answer isn’t clear. Although a large literature documents how people infer current dispositions from past behavior [1–3]—including inferences specifically about moral character [4–6]—much less is known about how moral inferences inform forecasts about the future. We present three studies that help fill this gap by testing three plausible moderators of the relationships between moral inferences and moral forecasts, including psychological properties of the person perceived (whether judgments were made about adults or children) and psychological properties of perceivers (belief in the stability of moral dispositions and belief in karma).

### A rocky road from dispositions to predictions?

Perceivers make dispositional inferences that serve a pragmatic purpose: They allow perceivers to forecast others’ actions in the immediate and long-term future [7–10]. These forecasts may be especially important in the moral domain. Inferences made from subjectively-appraised good and bad actions inform highly consequential interpersonal decisions—such as who to cooperate with and who should be avoided [11]. Moral inferences not only inform forecasts about a person’s future actions, but might also inform forecasts about their future outcomes: People who perform good behaviors may be expected to accrue the goodwill of others, enhancing the likelihood of future positive outcomes; whereas people who commit transgressions may experience retribution, social exclusion, and other future misfortunes, consistent with widespread intuitions about living in a just world [12–14].

Jones [15] characterized the process of inferring dispositions from actions as a “rocky road,” due to the many contextual variables that moderate perceivers’ tendency to draw correspondent inferences from a person’s actions. So too it may be a rocky road from inferences about a person’s current moral character to forecasts about their future character and future outcomes. Ample evidence of lay dispositionalism [2] suggests that perceivers often assume that current behavioural dispositions are diagnostic of future dispositions and experiences (and confirmation bias in processing new information can reinforce these expectations; [16]). But forecasts about the future depend tacitly on underlying assumptions about causality—including temporal consistency in behavioural dispositions and the expectation of immanent justice—which suggest specific variables that might moderate the extent to which perceivers use moral inferences to forecast the future.

#### Forecasts about adults and about children

Compared to adults, young children are perceived as less capable in the moral domain—less able to foresee the consequences of their actions, less able to control their own behavior, and thus less able to distinguish right from wrong [17, 18]. This difference impacts judgments about the perceived moral wrongness of adults’ and children’s transgressions, and impacts how these judgments are influenced by other variables (e.g., intentionality has weaker effects on judgments about children than about adults; [18]). Although previously unexplored, this difference may also influence forecasts about adults’ and children’s future moral character and future outcomes. The present studies provided descriptions of children and adults who engage in identical immoral actions, and asked participants to evaluate the transgressors’ current character traits, predict their future moral dispositions, and forecast their likelihood of experiencing various misfortunes in the future. If children (relative to adults) are perceived to have less fully-developed moral agency, then inferences about children’s moral character may be perceived to be relatively less diagnostic of their future moral character, and also less diagnostic of their future outcomes. The empirical implication is that correlations between moral inferences and moral forecasts are likely to be less strong when perceivers make judgments about children (compared to comparable contexts in which they make judgments about adults).

#### Individual differences in implicit personality theories

Individuals differ in the extent to which they believe that dispositions are stable across time (an “entity theory” of personality) or changeable across time (an “incremental theory” of personality), and these individual differences can impact moral judgments [19]. Compared to entity theorists, incremental theorists are less inclined to attribute transgressive behavior to stable dispositions, and respond less punitively to those transgressions [20, 21]. Not directly tested in previous research is the possibility that these implicit personality theories might also influence forecasts about others’ future moral dispositions. If perceivers endorse an incremental (rather than entity) theory of personality, then inferences about current moral character may be perceived to be less diagnostic of future character. The empirical implication is that the correlation between inferences about current moral character and forecasts about future moral character is likely to be less strong among perceivers who endorse an incremental theory of personality.

#### Individual differences in belief in karma

People differ in their beliefs about immanent justice—whether they believe that, in the long run, good people experience good fortune and bad people experience misfortune. Ample evidence suggests that these beliefs influence retrospective explanations for others’ current misfortunes, such that a person’s current successes or misfortunes are used to infer their likelihood of past virtuous or immoral actions [12–14]. These beliefs may also influence whether perceivers forecast that future outcomes will be consistent with a person’s current moral character. To explore this possibility, we drew upon, and sought to extend, psychological research on the belief in *karma*—a supposed supernatural source of moral justice [22].

Belief in karma is prevalent even among people with little or no cultural exposure to karmic theological teachings, it is distinct from beliefs about secular sources of immanent justice. Karma is conceptually distinct from other types of justice in that it ensures that good and bad fortune befall those who deserve it even across long time scales that cannot plausibly be policed by human agents (e.g., across otherwise unrelated situations, or across reincarnations into a new body), and belief in karma is only moderately correlated with individual differences in other fairness and justice beliefs (e.g., belief in a just world). Belief in karma also uniquely predicts the attribution that current misfortunes as caused by past bad deeds, even after controlling for secular justice beliefs [23]. Just as belief in karma impacts attributions about an individual’s past misfortunes, it is likely to also influence forecasts about future outcomes: Among people who more strongly believe in karma, judgments about a person’s current goodness or badness may be perceived as more diagnostic of that person’s future good or bad outcomes. The empirical implication is that the correlation between inferences about current moral character and forecasts about future outcomes is likely to be stronger among people who more strongly believe in karma.

We tested whether karmic beliefs moderate forecasts about two different types of events: (a) Interpersonal misfortunes, events that are explicitly caused by the actions of other people (e.g., being cheated on or stolen from) and therefore can be explained by mundane social causes (i.e., the expectation that those with poor character traits will be treated unkindly in turn by the people around them); (b) accidental misfortunes that cannot be easily explained by mundane social or physical forces elicited by a target’s moral character (e.g., getting injured in a car accident or getting a serious illness). Accidental misfortunes are likely to be uniquely moderated by belief in karma, due to the supernatural element of karma that ensures moral justice even when no human agents are involved [23], whereas the more general, more secular belief in a just world might be less relevant to forecasts about accidental misfortunes.

### Overview of current research

Three studies investigated how features of the perceiver and the perceived moderate the strength of the relationship between inferences about a person’s current moral character (inferred from vignettes depicting specific actions) and forecasts about that person’s future moral character and future outcomes. In each study we manipulated whether transgressions were performed by an adult or child, and measured inferences about their current moral character, probable future moral character, and likelihood of experiencing various misfortunes. We assessed whether relationships between inferences and forecasts were moderated by the target’s age, and whether they were moderated by participants’ implicit personality theories and their belief in karma (measured as individual differences).

Each study assessed ratings of a variety of moral transgressions that vary in their intent to cause harm and the severity of the harmful consequences—variables which produce variation in the severity of moral evaluations transgressions [e.g., 24–27], and which we therefore expected to produce variance in current character inferences. This set of moral transgressions allowed us to test the replicability of our primary analyses across both scenarios where immoral character traits could be inferred from the transgressors’ intentional action (harmful, helpful, or neutral) (Studies 1 and 2), and scenarios where the transgressors’ harmful intentions were held constant (Study 3). Our primary analyses reported below focus on how moral character inferences, derived from these scenarios, were used by participants to forecast the transgressor’s future moral character and future life outcomes, and what variables moderate the strength of the relation between inferences and forecasts. Finally, we conducted an internal meta-analysis, to assess the overall pattern of effects across all studies. All studies were run concurrently in February and March of 2018.

Hypotheses, methods, and analysis plans were preregistered on the Open Science Framework prior to data collection (https://osf.io/u5gde/?view_only=f8ceb52bb92d4749885b551b7def755d). In the Methods and Results sections below (or in accompanying S1 File) we report all manipulations and measures completed by participants, as well as preregistered sample sizes and preregistered data exclusion decision-rules. In addition, we identify any deviations from preregistered analysis plans. All data is available at https://osf.io/9taex/?view_only=8e6e6f76ccc24035bf68381126bbdfd0.

## Study 1

### Methods

Procedures for all studies were approved by the Behavioural Research Ethics Board at the University of British Columbia (#H11-02441), and all participants provided written consent.

#### Participants

As preregistered, we aimed to recruit a sample of approximately 300 participants through Amazon’s Mechanical Turk (MTurk). This sample size would have 80% power to detect reasonably small correlations (*r* = .16) and within-subjects differences (*d* = 0.16). Based on power analyses conducted via simulation [28], this sample size has approximately 80% power to detect interactions of *b* = 0.18, which is smaller than previously-observed interactions between transgressor age and other morally-relevant variables (e.g., intentionality and disgustingness of actions, *b >* 0.26, [18]), between belief in karma and other factors of moral judgements (e.g., valence of past behavior, *b >* .20, [23]), and between entity theorists and incremental theorists’ judgments of children’s transgressions (e.g., *η*_*p*_^2^ = 0.03, 21). Based on preregistered criteria, we excluded 30 participants who failed at least one of two attention check questions, resulting in a final sample size of 299. 42% of participants reported being parents (exploratory analyses indicated that parental status did not significantly moderate any of the effects reported below). All sample demographics are provided in S1 File.

#### Vignettes describing moral transgressions

After giving written informed consent, participants read four vignettes that each described an event in which one individual (the transgressor) caused harm to another person (adapted from [18], Study 3). For example, participants read that “Two men, Alex and Mike, were at a party. There were lots of people there, and everyone was having a good time. It was very crowded and there was not very much room to walk through the crowd. Alex was sitting down and suddenly Mike turned and intentionally struck Alex in the face with his hand, severely bruising his eye.” Within this set of four vignettes, we orthogonally manipulated (1) whether the harmful action was intentional (e.g., “Mike turned and intentionally struck Alex in the face”) or accidental (e.g., “Mike tripped and fell forward and his hand accidentally struck Alex in the face”), to ensure that there was sufficient variability in moral character inferences (across participants and across targets) to use as a predictor of future forecasts, and (2) whether the transgressor was an adult (e.g., “an adult man”) or child (e.g., “a 3-year old boy”). Each vignette referred to a different transgressor (with name, age, and gender varied across vignettes) who performed a different action (hitting someone, cutting someone with shattered glass, not warning someone about a dangerous situation, making someone have an allergic reaction). Vignette presentation order was randomized and specific vignette content matched with each condition (i.e., adult/intentional, adult/accidental, child/intentional, child/accidental) was counterbalanced across participants. (All materials, used in all studies, are available in the preregistration documents).

#### Moral inferences and moral forecasts

After each vignette, participants provided various judgments of about the transgressor. All responses were made on 7-point scales. Mean composite scores were computed for each measure, after reverse-scoring specific items so that higher values indicated more negative evaluations.

*Moral wrongness of the action*. Four items (α = .94) assessed judgments about the extent which the transgressor’s behavior was “bad”, “unacceptable”, should be “punished,” or should be “forgiven.”

*Transgressor’s current moral character*. Four items assessed judgments about the transgressor’s current dispositional tendency to be “kind,” “fair,” “dishonest,” and “selfish”; four additional items assessed judgments about the likelihood that, within the next month, the transgressor would “harm other people,” “lie to other people, “help others who are in need,” and “share with others.” Although superficially distinct, indices of trait ratings and action likelihoods were very highly correlated (*r* = .88, 95% CI [.86, .89]) across all targets, although the correlation was slightly smaller when evaluating child transgressors (*r* = .84) than adult transgressors (*r* = .91), showing preliminary evidence that inferences about children’s character are less consistent than inferences about adults’ character. All eight items were combined into a single composite index (α = .94). Results showed the same pattern of findings (in both magnitude and statistical significance) when using only the four character trait items, dropping the action likelihood items, which could also be conceived of as a measure of the transgressor’s future character, albeit in the very near future (the next month) rather than the more distant future (20 years from now, as measured in the future character measure).

*Transgressor’s future moral character*. An index comprised of 16 items assessed forecasts about the transgressor’s traits and actions “20 years from now” (α = .98.). We chose this timeframe to ensure that all targets would be adults by this future timepoint, even the targets who were currently children in the vignette descriptions, maximizing the opportunity for participants to report that moral character can change across different life stages. Eight items were identical to those used to assess inferences about current moral character (see above); 8 additional items assessed forecasts about additional morality-relevant traits (“cooperative,” “compassionate,” “unprincipled,” “irresponsible”) and actions (“cheat on a test,” “betray their friends and family members,” “refuse to give to charity, even when they have enough money,” “talk about others behind their backs”). Results showed the same pattern of findings (in both magnitude and statistical significance) when using the full 16-item measure of moral character, and when using an 8-item measure of future moral character that drops the items that are duplicated in the current moral character and future moral character measures. The measure of future moral character was also very highly correlated with the measure of current moral character, *r* = .91 95% CI [.90, .92]. Despite this high correlation, the analyses below focus on separate composite measures of current character and future character inferences (while aggregating across inferences and traits within each timepoint) as the most parsimonious way of testing our theoretically-derived hypotheses while encompassing both trait and action likelihood measures. (When analyzed separately, the two different indicators of character that show similarly-sized differences over time according to the transgressor’s age).

*Transgressor’s future misfortunes*. Participants forecast the likelihood that the transgressor would experience various negative outcomes “at some point in the future.” Five items referred to misfortunes resulting from actions taken by other people (“treated rudely by other people,” “betrayed by a friend,” “fired from [his/her] job,” “cheated on by a romantic partner,” and “have something valuable stolen”). Three other items referred to non-interpersonal, accidental misfortunes (“get injured in a car accident,” “have [his/her] home damaged by a natural disaster (e.g., hurricane, fire),” and “get a serious illness that requires [him/her] to go to the hospital.”) For all studies we conducted separate analyses on indices assessing forecasts about *future interpersonal misfortunes* (α = .91) and *future accidental misfortunes* (α = .89). All of these items refer to somewhat common bad experiences that could befall any person, and include some experiences that may plausibly be caused by one’s own or another person’s actions, and some experiences that are more plausibly the result of natural circumstances beyond one’s control. These forecasts have conceptually distinct associations with moral character—interpersonal misfortunes (more so than accidental misfortunes) can be interpreted as the result of moral character via naturalistic (rather than supernatural) causal pathways—and were also empirically distinct: An exploratory factor analysis indicated that a one-factor solution was not a good fit for the data, *RMSEA* = .27, 90% CI [.26, .28], but a two-factor model was a good fit, *RMSEA* = .061 [.047, .075], and revealed two moderately correlated factors, *r* = .49, with no large cross-loading (all < .27). Accidental misfortunes were also judged far less likely overall than were interpersonal misfortunes, *d* = 0.66, p < .001. We therefore present separate analyses for forecasts about interpersonal and accidental misfortunes in all studies.

#### Individual difference measures

In addition to a questionnaire assessing demographic characteristics (e.g., gender, age, religious affiliation), participants completed the following individual difference measures (additional questionnaires, not relevant to present analyses, are reported in the preregistration documents).

*Implicit personality theory (of moral character)*. An 8-item index ([29]; adapted from items previously used by [19]; α = .96) assessed the extent to which participants believed that a person’s moral character is either stable or changeable over time (e.g., “A person’s moral character is something very basic about them and it can’t be changed much”). Higher values indicate greater perceived stability.

*Belief in karma*. Belief in karma was assessed with a 16-item questionnaire ([23]; α = .94) that has been validated across multiple cultural populations varying in ethnicity and religious beliefs. (Sample items: “When people are met with misfortune, they have brought it upon themselves by behavior in a past life”; “When someone does a good deed, even if there are no immediate consequences, they will be rewarded for it in some future time in their life.”) Higher values indicate stronger belief in karma.

*Belief in a just world*. Participants also completed an 8-item measure that assesses belief in a just world, the expectation of fair treatment within one’s life [30] (α = .89). This scale does not refer to any supernatural forces, and was included to explore whether forecasts of the future were moderated by beliefs about justice/fairness in general, or uniquely moderated by belief in karma, due to the expression of karmic causality over long timeframes (beyond that policed by secular sources of justice).

### Results

#### Analysis strategy

Preliminary analyses assessed whether inferences about current moral character sensibly reflect features of the vignettes (e.g., intentional harms are worse than accidental harms). Primary analyses then examined relations between inferences about current character and forecasts about future character and future misfortunes, and tested hypothesized moderators. (Full models and alternative analyses are presented in the S1 File.) Analyses were performed as mixed-effects models using the *lme4* package in R, including random intercepts and (when possible) slopes for moral character inferences nested within participant, to account for repeated measures (as a robustness check, we also conducted exploratory analyses that only analyzed the first vignette presented to participants; there was no evidence that trial order had an effect on responses, and this analysis produced similar effect sizes across studies to those presented below in our main preregistered analyses). Manipulated variables were dummy coded (transgressor age: 0 = child, 1 = adult; intentionality: 0 = accidental, 1 = intentional), and moral judgments and individual difference measures were standardized prior to analysis.

#### Inferences about current moral character

Preliminary analyses confirmed that intentional transgressions, compared to accidental transgressions, were judged to be more wrong, *b* = 2.93 95% CI [2.75, 3.12], and resulted in more negative inferences about current moral character, *b* = 1.14 [1.03, 1.25]. The effect of intentionality on wrongness and character inferences was weaker for child transgressors than adult transgressors (interaction *b*’s = 0.71 [0.45, 0.97] and 0.59 [0.44, 0.74], respectively; *p*’s < .001). The relationship between wrongness judgments and moral character inferences was also weaker for child transgressors (interaction *b* = 0.12 [0.07, 0.17], *p* < .001). These results replicate previous findings [18]), and show that the intentionality manipulation produced meaningful variability in current moral character inferences, allowing us to test the relationship between inferences and moral forecasts.

Additional pre-registered analyses examined ratings of the transgressors’ mental capabilities (including morally-relevant agentic capabilities, non-moral agentic capabilities, and affective capabilities). Across all studies, more negative judgments of wrongness and moral character were associated with ratings of lower mental capabilities. This was the case for judgments about adults (consistent with previous research [5, 31] and also judgments about children (see S1 File for full details).

#### Forecasts about future moral character

There was a very strong positive relationship between inferences about transgressors’ current moral character and forecasts about their future character, *r* = .91 95% CI [.90, .92]. Did the size of this relationship depend on whether the transgressor was an adult or child? Yes. Mixed effects models revealed both a main effect of transgressor’s age, and a significant interaction (Table 1): Although the relationship between inferences and forecasts was strong under all experimental conditions, it was less strong when the transgressor was a child.

**Table 1:** Forecasts about future moral character predicted by (a) inferences about current moral character, (b) target age (adult or child), and (c) the interaction between current moral character and target age.

	Study 1			Study 2			Study 3A			Study 3B		
	*b* [95% CI]	*SE*	*p*	*b* [95% CI]	*SE*	*p*	*b* [95% CI]	*SE*	*p*	*b* [95% CI]	*SE*	*p*
Intercept	-0.09 [-0.14, -0.04]	0.03	**< .001**	-0.06 [-0.11, -0.01]	0.03	**.025**	-0.15 [-0.23, -0.08]	0.04	**< .001**	-0.14 [-0.23, -0.06]	0.04	**.001**
Current Moral Character	0.82 [0.79, 0.86]	0.02	**< .001**	0.78 [0.74, 0.82]	0.02	**< .001**	0.83 [0.77, 0.89]	0.03	**< .001**	0.80 [0.73, 0.88]	0.04	**< .001**
Target Age (Adult or Child)	0.17 [0.11, 0.24]	0.03	**< .001**	0.11 [0.06, 0.17]	0.03	**< .001**	0.24 [0.15, 0.32]	0.04	**< .001**	0.18 [0.10, 0.26]	0.04	**< .001**
Current Moral Character × Target Age	0.06 [0.01, 0.10]	0.02	**.010**	0.06 [0.01, 0.10]	0.02	**.008**	0.05 [-0.03, 0.12]	0.04	.25	0.02 [-0.06, 0.11]	0.04	.56
Marginal R^2^/ Conditional R^2^	.831 / .856			.768 / .855			.602 / .781			.572 / .797		

Contrary to hypotheses, there was no evidence that the size of this relationship was moderated by participants’ implicit theories about the stability of moral character (Table 2). Exploratory analyses indicated that individual differences in implicit personality theories also did not moderate the association between judgments about moral wrongness and inferences about current moral character, *b* = 0.01 [-0.02, 0.04], *p* = .60. Nor did any such interaction emerge in either Study 2 (*b* = 0.01 [-0.01, 0.03], *p* = .33) or Study 3a (*b* = -0.02 [-0.05, 0.02], *p* = .36). Adding transgressor age as an additional covariate did not change these null findings, in any study.

**Table 2:** Forecasts about future moral character predicted by (a) inferences about current moral character, (b) individual differences in implicit personality theories, and (c) the interaction between current moral character and implicit personality theories.

	Study 1			Study 2			Study 3A		
	*b* [95% CI]	*SE*	*p*	*b* [95% CI]	*SE*	*p*	*b* [95% CI]	*SE*	*p*
Intercept	-0.00 [-0.04, 0.04]	0.02	.94	0.00 [-0.04, 0.04]	0.02	.99	-0.02 [-0.09, 0.04]	0.03	.47
Current Moral Character	0.86 [0.84, 0.89]	0.01	**< .001**	0.82 [0.80, 0.85]	0.01	**< .001**	0.91 [0.86, 0.96]	0.03	**< .001**
Implicit Personality Theories	-0.00 [-0.04, 0.04]	0.02	.94	0.01 [-0.04, 0.05]	0.02	.77	0.08 [0.02, 0.14]	0.03	**.014**
Current Moral Character × Implicit Personality Theories	0.01 [-0.01, 0.03]	0.01	.34	-0.00 [-0.03, 0.02]	0.01	.92	-0.02 [-0.07, 0.03]	0.03	.42
Marginal R^2^/ Conditional R^2^	.828 / .849			.768 / .852			.609 / .779		

#### Forecasts about future misfortunes

There was a positive relationship between inferences of current moral character and forecasts about future interpersonal misfortunes, and (to a lesser extent) forecasts about future accidental misfortunes (Table 3). Did the size of these relationships depend on whether the transgressor was an adult or child? Mixed effects models (Table 3) revealed no such moderation for forecasts about accidental misfortunes, but there was evidence of statistically significant moderation for forecasts about interpersonal misfortunes: Current character inferences were less strongly associated with forecasts about interpersonal misfortunes when the transgressor was a child, *b* = 0.35 [0.30, 0.40], *p* < .001, rather than an adult, *b* = 0.42 [0.37, 0.46], *p* < .001.

**Table 3:** Forecasts about future misfortunes (interpersonal and accidental) predicted by (a) inferences about current moral character, (b) target age (adult or child), and (c) the interaction between current moral character and target age.

	Study 1			Study 2			Study 3A			Study 3B		
	Interpersonal Misfortunes											
	*b* [95% CI]	*SE*	*p*	*b* [95% CI]	*SE*	*p*	*b* [95% CI]	*SE*	*p*	*b* [95% CI]	*SE*	*p*
Intercept	-0.03 [-0.14, 0.08]	0.06	.56	-0.04 [-0.12, 0.04]	0.04	.35	-0.09 [-0.19, 0.01]	0.05	.087	-0.07 [-0.19, 0.06]	0.07	.31
Current Moral Character	0.35 [0.30, 0.40]	0.03	**< .001**	0.29 [0.23, 0.34]	0.03	**< .001**	0.32 [0.25, 0.39]	0.04	**< .001**	0.36 [0.27, 0.44]	0.04	**< .001**
Target Age (Adult or Child)	0.07 [0.00, 0.14]	0.03	**.049**	0.07 [0.00, 0.14]	0.03	**.036**	0.11 [0.03, 0.20]	0.04	**.009**	0.10 [0.01, 0.20]	0.05	**.031**
Current Moral Character × Target Age	0.07 [0.02, 0.12]	0.02	**.007**	0.09 [0.04, 0.14]	0.03	**< .001**	0.10 [0.02, 0.18]	0.04	**.016**	0.01 [-0.09, 0.10]	0.05	.89
Marginal R^2^/ Conditional R^2^	.226 / .789			.187 / .728			.155 / .684			.127 / .715		
	Accidental Misfortunes											
	*b [95% CI]*	*SE*	*p*	*b [95% CI]*	*SE*	*p*	*b [95% CI]*	*SE*	*p*	*b [95% CI]*	*SE*	*p*
Intercept	0.00 [-0.13, 0.13]	0.06	1.00	-0.00 [-0.09, 0.08]	0.04	.93	-0.03 [-0.14, 0.09]	0.06	.66	-0.01 [-0.16, 0.14]	0.08	.89
Current Moral Character	0.09 [0.06, 0.13]	0.02	**< .001**	0.09 [0.04, 0.14]	0.02	**< .001**	0.10 [0.05, 0.15]	0.03	**< .001**	0.18 [0.11, 0.25]	0.04	**< .001**
Target Age (Adult or Child)	0.01 [-0.04, 0.07]	0.03	.64	0.01 [-0.04, 0.06]	0.03	.73	0.02 [-0.04, 0.09]	0.03	.46	0.02 [-0.07, 0.10]	0.04	.68
Current Moral Character × Target Age	0.03 [-0.01, 0.08]	0.02	.095	-0.02 [-0.06, 0.02]	0.02	.44	0.03 [-0.03, 0.10]	0.03	.29	-0.06 [-0.15, 0.02]	0.04	.14
Marginal R^2^/ Conditional R^2^	.022 / .836			.010 / .813			.015 / .819			.020 / .792		

Did belief in karma moderate the size of these relationships? The results differed depending on the kind of misfortune: A stronger belief in karma predicted a significantly stronger relation between current character inferences and forecasts of accidental misfortunes; but for forecasts of interpersonal misfortunes the moderating effect was only marginally significant (Table 4). Additional exploratory analyses revealed that belief in a just world—a variable that does not necessarily entail justice outside the bounds of mundane physical/social causality, and it only weakly correlated with belief in karma, *r* = .24 [.13, .34], *p* < .001—did not significantly moderate the size of either relation (interpersonal misfortunes: *b* = 0.03 [-0.01, 0.06], *p* = .15; accidental misfortunes: *b* = -0.01 [-0.03, 0.02], *p* = .72).

**Table 4:** Forecasts about future misfortunes (interpersonal and accidental) predicted by (a) inferences about current moral character, (b) individual differences in belief in Karma, and (c) the interaction between current moral character and belief in Karma.

	Study 1			Study 2			Study 3A			Study 3B		
	Interpersonal Misfortunes											
	*b* [95% CI]	*SE*	*p*	*b* [95% CI]	*SE*	*p*	*b* [95% CI]	*SE*	*p*	*b* [95% CI]	*SE*	*p*
Intercept	0.01 [-0.10, 0.11]	0.05	.91	0.00 [-0.07, 0.08]	0.04	.91	-0.01 [-0.10, 0.08]	0.05	.83	-0.01 [-0.12, 0.11]	0.06	.92
Current Moral Character	0.40 [0.36, 0.44]	0.02	**< .001**	0.35 [0.31, 0.39]	0.02	**< .001**	0.39 [0.34, 0.45]	0.03	**< .001**	0.39 [0.32, 0.45]	0.04	**< .001**
Belief in Karma	0.05 [-0.06, 0.15]	0.05	.40	-0.00 [-0.08, 0.07]	0.04	.93	0.08 [-0.01, 0.17]	0.05	.068	0.19 [0.08, 0.31]	0.06	**.001**
Current Moral Character × Belief in Karma	0.04 [-0.00, 0.08]	0.02	.061	0.05 [0.01, 0.09]	0.02	**.012**	0.00 [-0.05, 0.06]	0.03	.89	0.13 [0.06, 0.20]	0.03	**< .001**
Marginal R^2^/ Conditional R^2^	.229 / .788			.198 / .726			.165 / .685			.174 / .716		
	Accidental Misfortunes											
	*b [95% CI]*	*SE*	*p*	*b [95% CI]*	*SE*	*p*	*b [95% CI]*	*SE*	*p*	*b [95% CI]*	*SE*	*P*
Intercept	0.01 [-0.11, 0.13]	0.06	.88	0.00 [-0.08, 0.09]	0.04	.94	-0.01 [-0.12, 0.10]	0.06	.90	-0.00 [-0.14, 0.14]	0.07	.95
Current Moral Character	0.12 [0.09, 0.15]	0.01	**< .001**	0.07 [0.04, 0.11]	0.02	**< .001**	0.12 [0.08, 0.16]	0.02	**< .001**	0.16 [0.10, 0.21]	0.03	**< .001**
Belief in Karma	0.12 [0.00, 0.25]	0.06	**.046**	0.04 [-0.04, 0.12]	0.04	.36	0.16 [0.05, 0.27]	0.06	**.005**	0.19 [0.05, 0.33]	0.07	**.007**
Current Moral Character × Belief in Karma	0.03 [0.00, 0.06]	0.01	**.044**	0.03 [-0.00, 0.07]	0.02	.060	0.03 [-0.02, 0.07]	0.02	.24	0.12 [0.07, 0.18]	0.03	**< .001**
Marginal R^2^/ Conditional R^2^	.034 / .836			.013 / .813			.036 / .820			.059 / .792		

Additional exploratory analyses tested whether participants’ implicit theories moderated relations between current moral character and forecasts of future misfortunes. There was no evidence of any such moderating effect, for either interpersonal outcomes, *b* = 0.01 [-0.02, 0.05], or accidental outcomes, *b* = 0.00 [-0.03, 0.03] (Studies 2 and 3 similarly found no moderating effects, see S1 File).

### Summary and discussion

Moral character inferences were associated (very strongly) with forecasts about their future character and also (less strongly) with forecasts about future misfortunes. As predicted, these relationships were somewhat weaker when the transgressor was a child, rather than an adult (although the relationship between current and future character was quite strong in both cases). These findings extend previous research showing differences in the extent to which specific variables predict moral judgments about children and about adults [18]. In contrast to past research and our hypotheses, we found no evidence that participants’ implicit theories of moral character moderated the relationship between moral inferences and moral forecasts: Predictions about future moral character did not differ between participants who reported that character is generally stable and those who reported that character is changeable over time.

We also found that, as hypothesized, individual differences in belief in karma moderated forecasts of the future: Belief in karma was associated with a somewhat greater perceived likelihood that future misfortunes would befall individuals who were judged to have bad moral character. These findings extend previous research showing that belief in karma uniquely predicts attributions for past misfortunes [23], to show a similar pattern in forecasts of the future.

## Study 2

Study 2 served as a conceptual replication of Study 1, using different vignettes and a different experimental manipulation to elicit variability in participants’ moral judgments: Participants responded to vignettes depicting actions that were either harmful, helpful, or were neither harmful nor helpful to another person.

### Methods

#### Participants

Following the same preregistered recruitment and data-exclusion criteria as in Study 1, 729 participants were recruited from MTurk and data from 69 participants were excluded prior to data analysis, leaving a final sample of 660. Given the addition of a between-subjects manipulation, we increased the planned sample size to 200 per condition to provide 80% power to detect small-to-medium between-condition differences (*d* = 0.28), in addition to retaining high (>90%) power to detect the small interaction effects (as predicted in Study 1).

#### Vignettes describing moral actions

After providing informed consent, participants read two vignettes, one describing an action performed by a child and one describing an action performed by an adult. Participants were randomly assigned to read vignettes describing either (a) an adult and a child who perform harmful actions (e.g., “Emily noticed Olivia drop the money on the floor, and Emily reached down to take the money for herself”), (b) an adult and a child who perform helpful actions (e.g., “Emily noticed Olivia drop the money on the floor, so Emily picked it up and returned it to Olivia”), or (c) an adult and a child who perform morally neutral actions that were neither helpful nor harmful (e.g., “Emily saw Olivia notice that she had dropped the money, and saw Olivia pick it back up”). This set of scenarios was chosen to elicit an even greater range of inferences about moral character than were available in Study 1, by including both intentionally harmful actions, that signal immoral character traits, and explicitly helpful prosocial actions, that signal especially positive moral character traits (not merely the less-strongly-negative evaluations that were inferred from accidental transgressions in Study 1). Each vignette referred to a different transgressor performing a different action (e.g., causing [or preventing] someone from getting hurt; taking [or returning] someone’s money; not warning [or warning] someone about a dangerous situation; giving someone food they are allergic to [or a food they really like]), vignette presentation order was randomized, and specific vignette content was counterbalanced across participants.

#### Moral inferences and moral forecasts

After reading each vignette, participants responded to measures that assessed the same judgments assessed in Study 1 (α’s ranged from .88 to .95). The specific sets of items included in each measure were identical to those in Study 1, with two exceptions. An item assessing forgiveness was omitted from the composite index assessing judgments of the moral wrongness. (Also, for this study only, the endpoints of the moral wrongness response scale were altered to accommodate praise for helpful actions as well as condemnation of hurtful actions.) The index assessing forecasts of future moral character was comprised of 8 items, rather than 16 items (we omitted the 8 items from the future character measure in Study 1 that duplicated the traits and action likelihoods described in the current character measure).

#### Individual difference measures

Participants completed the same individual difference measures assessed in Study 1.

### Results

#### Inferences about current moral character

Preliminary analyses confirmed that harmful actions were judged to be more wrong than neutral actions (*b* = 2.71 [2.48, 2.94]) and resulted in more negative inferences about current moral character (*b* = 0.66 [0.54, 0.78]). Interactions with target age revealed that these effects were significantly weaker when judging children rather than adults (interaction *b* = 0.99 [0.72, 1.25] and *b* = 0.73 [0.60, 0.86], for judgments of moral wrongness and inferences about current character, respectively). Helpful actions were evaluated more positively than neutral actions (*b* = -2.77 [-3.00, -2.55]) and resulted in more positive inferences about current moral character (*b* = -0.80 [-0.92, 0.69]); the sizes of these effects were not significantly moderated by target age (interactions *b*’s = 0.21 [-0.05, 0.48] and = -0.02 [-0.14, 0.10], *p* = .11 and .82, respectively). The relationship between wrongness judgments and moral character inferences was weaker when evaluating children than when evaluating adults (interaction *b* = 0.11 [0.08, 0.14], *p* < .001). These results again document meaningful variability in moral character inferences, which allows us to test the relations between these inferences and moral forecasts.

#### Forecasts about future moral character

Replicating Study 1, inferences about current moral character strongly predicted forecasts of future moral character, but—as indicated by a statistically significant interaction with target age (Table 1)—this relation was somewhat less strong when the target of judgment was a child (*b* = 0.78 [0.74, 0.82]) rather than an adult (*b* = 0.84 [0.81, 0.87]). The size of this relation was not moderated by individual differences in implicit personality theories of moral character (Table 2).

#### Forecasts about future misfortunes

Inferences about current moral character were positively associated with forecasts about interpersonal misfortunes and (more weakly) with forecasts about accidental misfortunes. Replicating the pattern found in Study 1, a statistically significant interaction with target age (Table 3) indicated that the relationship with interpersonal misfortunes (but not accidental misfortunes) was weaker when the target was a child (*b* = 0.29 [0.23, 0.34]) rather than an adult (*b* = 0.38 [0.33, 0.42]).

Stronger belief in karma predicted a significantly stronger relation between current character inferences and forecasts of interpersonal misfortunes; but for forecasts of accidental misfortunes the moderating effect was only marginally significant (although of a similar magnitude to the effects observed in Study 1, Table 4). Belief in a just world also moderated the size of the relation with forecasts of interpersonal misfortunes (*b* = 0.05 [0.01, 0.09], *p* = .009) but not the relation with forecasts of accidental misfortunes (b = -0.00 [-0.04, 0.03], *p* = .91).

### Summary and discussion

Study 2 produced patterns of results similar to those observed in Study 1. Current moral character inferences very strongly predicted forecasts about future character, less strongly predicted forecasts about future interpersonal misfortunes, and even less strongly—but still positively—predicted forecasts about future accidental misfortunes. As hypothesized, the magnitude of character inferences’ relations with forecasts about future moral character, and future interpersonal misfortunes, were less strong when evaluating children. The magnitude of the relation with forecasts about misfortunes was also moderated (significantly, for interpersonal misfortunes, and marginally, for accidental misfortunes) by belief in karma: Negative moral character evaluations predicted a greater likelihood of negative future outcomes among participants with stronger beliefs in karma. Also consistent with Study 1, there was no evidence of any moderating effect of perceivers’ implicit personality theories, thus further failing to support our hypothesis that implicit theories will moderate moral forecasts.

## Study 3

Study 3 provided additional tests of the primary hypotheses, within a design that included another experimental manipulation to elicit variability in participants’ moral judgments. All vignettes described transgressors who engaged in actions with harmful intent; in some versions of these vignettes the transgressor’s actions actually had harmful consequences, whereas in other versions there were no harmful consequences.

We created two versions of Study 3, which varied the order in which materials were presented to participants. In Study 3A, participants completed all individual difference measures at the end of the procedures (consistent with the procedure used in Studies 1 and 2). In Study 3B, participants completed the belief in karma questionnaire first—a procedural change designed to make participants’ karmic beliefs more salient while responding to vignettes. Study 3A was preregistered, and Study 3B was not, but identical participant recruitment criteria and analyses were used in both samples, and data was collected concurrently.

### Study 3A

#### Methods

*Participants*. Following the same preregistered criteria identified in Study 1, 327 participants were recruited from MTurk and data from 18 participants were excluded prior to data analysis, leaving a final sample of 309.

*Materials*. After providing informed consent, participants read four vignettes, each of which described an event in which one individual intended to cause harm to another person (e.g., “Megan gave Kate a peanut-butter cookie to eat. Megan thinks that Kate is allergic to peanut butter, but intentionally gave her the cookie anyways”). We orthogonally manipulated two variables within this set of four vignettes: The transgression had either a harmful outcome (e.g., “Kate had a severe allergic reaction from eating the cookie”) or a non-harmful outcome (e.g., “Kate is actually allergic to almonds, not peanuts, and she was not affected by eating the cookie”); and the transgressor was described as either an adult or a child. These scenarios allowed us to test our hypotheses in a context where all targets had potentially harmful intentions that could lead to inferences of immoral character traits, while retaining variability in evaluations across participants and across different targets (i.e., intentionally harmful actions tend to be judged more negatively when they cause more harmful outcomes, e.g., [27]). Each vignette referred to a different transgressor performing a different action (e.g., succeeding or failing to cut someone with broken glass; succeeding or failing to take money from a stranger; not warning someone about a dangerous situation that causes an injury or does not cause an injury; succeeding or failing to give someone an allergic reaction), vignette presentation order was randomized, and specific vignette content was counterbalanced across participants. After reading each vignette, participants responded to items that provided the composite indices of moral inferences and moral forecasts assessed in Study 2 (α’s ranged from .86 to .93). Finally, participants completed the same individual difference measures as in Studies 1 and 2.

### Results

#### Inferences about current moral character

Preliminary analyses confirmed that actions with harmful outcomes (compared to identical actions with non-harmful outcomes) were judged to be more wrong (*b* = 0.68 [0.49, 0.88]) and resulted in more negative inferences about current moral character (*b* = 0.13 [0.02, 0.25]). Transgressor’s age (adult or child) did not moderate the effect of harm on either wrongness judgments or moral character inferences (interaction *b’s* = -0.12 [-0.40, 0.16] and *b* = 0.01 [-0.15, 0.17], respectively; *p’s* = .39 and .92), nor did it moderate the association between wrongness judgments and character inferences (*b* = -0.03 [-0.09, 0.03], *p* = .40).

#### Forecasts about future moral character

Mixed effects models revealed that inferences about current moral character were very strongly associated with forecasts about future moral character, and that—although there were main effects of transgressor’s age and participants’ implicit personality theories—neither of those variables moderated this relationship between inferences and forecasts about moral character (see Tables 1 and 2).

#### Forecasts about future misfortunes

Inferences about transgressor’s current moral character were associated with forecasts about future interpersonal misfortunes and (more weakly) forecasts about accidental misfortunes. As in Studies 1 and 2, a significant interaction with transgressor age (Table 3) indicated that the relationship with interpersonal misfortunes (but not accidental misfortunes) was weaker when the transgressor was a child (*b* = 0.32 [0.25, 0.39]) rather than an adult (*b* = 0.42 [0.34, 0.50]). Results also show that, although there was a main effect of belief in karma on forecasts about future misfortunes, belief in karma did not moderate the relationship between inferences about current moral character and forecasts about future misfortunes (thus failing to replicate the previous two studies, although the magnitude of the moderation of accidental misfortunes was consistent across all studies, Table 4). Belief in a just world did moderate the relationship between character inferences and forecasts about interpersonal misfortunes (*b* = 0.06 [0.00, 0.11], *p* = .033), but not accidental misfortunes (*b* = .02 [-0.02, 0.06], *p* = .38).

### Study 3B

#### Methods

230 participants were recruited from MTurk (this sample size is smaller than that in Study 3A due to an oversight that occurred during data collection). After excluding 12 participants (according to criteria preregistered for Study 3A), there was a final sample of 218 participants. The methods were identical to the methods of Study 3A, except for two elements. Participants completed the belief in karma questionnaire prior to completing all other measures—a procedure designed to bolster the psychological salience of participants’ beliefs about karma while completing the moral judgment tasks. Due to the small moderation by belief in karma observed in Study 1, we suspected that the moderating effect would be larger and/or more robust if karma beliefs were salient to participants. And, for the sake of procedural brevity, no additional individual difference measures were assessed (i.e., we did not to include measures of belief in a just world or implicit personality theories).

### Results

#### Inferences about current moral character

Preliminary analyses again confirmed that actions with harmful outcomes were judged to be more wrong and resulted in more negative inferences about current moral character (*b* = 0.44 [0.21, 0.67] and *b* = 0.14 [0.00, 0.29], respectively; *p’s* < .001 and .045). There was again no evidence that inferences about current character were moderated by transgressor’s age, *b* = 0.10 [-0.10, 0.30], *p* = .33.

#### Forecasts about future moral character

Inferences about current moral character very strongly predicted forecasts about future moral character, and as in Study 3A there was no evidence that this effect was moderated by transgressor’s age (see Table 1).

#### Forecasts about future misfortunes

Inferences about transgressor’s current moral character were positively related to forecasts about future interpersonal misfortunes and (more weakly) forecasts about accidental misfortunes. There was no evidence that these relations were meaningfully moderated by transgressor’s age (Table 3). In contrast, statistically significant interactions (Table 4) showed that these relations were moderated by belief in karma: Among participants who more strongly believed in karma, inferences about current moral character were more strongly associated with forecasts about both interpersonal and accidental misfortunes.

### Summary and discussion

Studies 3A and 3B confirmed that inferences about current moral character strongly inform forecasts about future character and also (less strongly) inform forecasts about future misfortunes. Unlike Studies 1 and 2, neither version of Study 3 revealed any moderating effect of transgressor’s age (adult or child) on the relation between current moral character and forecasts about future moral character. But Study 3A did replicate a moderating effect of transgressor’s age on the relation with forecasts about future interpersonal misfortunes. Study 3B replicated the moderating effect of belief in karma, which was associated with a stronger relationship between inferences about moral character and forecasts about future misfortunes. In general, belief in karma had stronger moderating effects in Study 3B than in any other study, perhaps due to a procedural change that made participants’ karmic beliefs salient prior to responding to the moral vignettes. (If there is merit to this explanation, it suggests that these moderating effects might generally be stronger within Hindu and Buddhist populations, in which beliefs about karma are reinforced by cultural factors and are likely to be more chronically accessible; 22). Finally, the results of Study 3 once again failed to reveal any moderating effect of participants’ implicit personality theories, thus providing consistent evidence across all studies that individual differences in implicit theories are unrelated to the relationship between moral inferences and moral forecasts.

## Meta-analytic summary

All studies showed that moral inferences predicted moral forecasts, and also provided some evidence of moderating variables. The magnitudes and statistical significance of these moderators varied across these studies (a plausible pattern even with real effects; [32]). Therefore, we conducted an internal meta-analysis (using the *metafor* package in R) that estimated the effects of the hypothesized moderators across all studies (full results are available in the S1 File).

**Fig 1:**
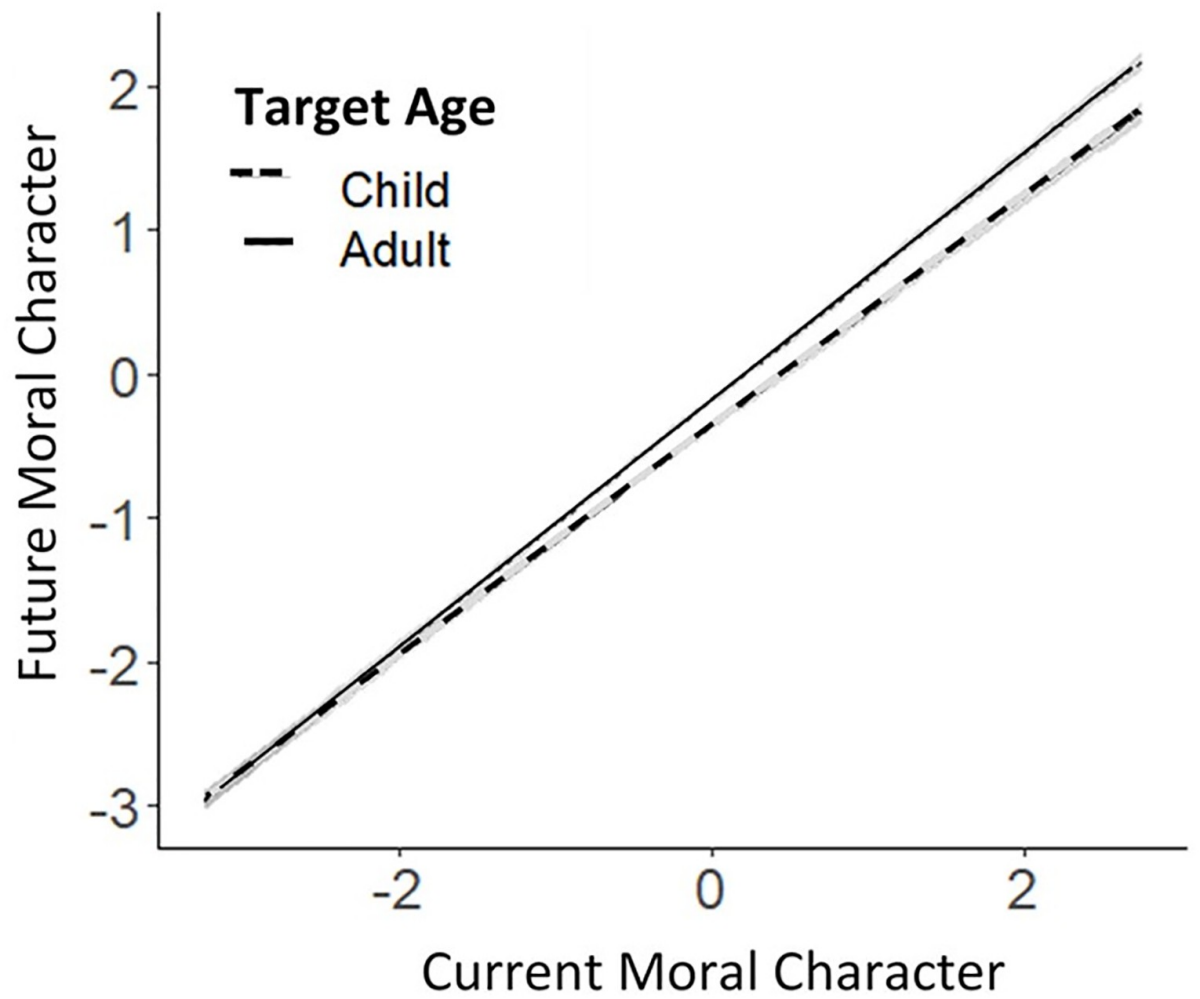
Regression, with 95% confidence bands, showing that the target’s age (adult or child) moderated the relationship between inferences about current moral character and forecasts about future moral character. Regression lines are plotted from data aggregated across all studies.

**Fig 2:**
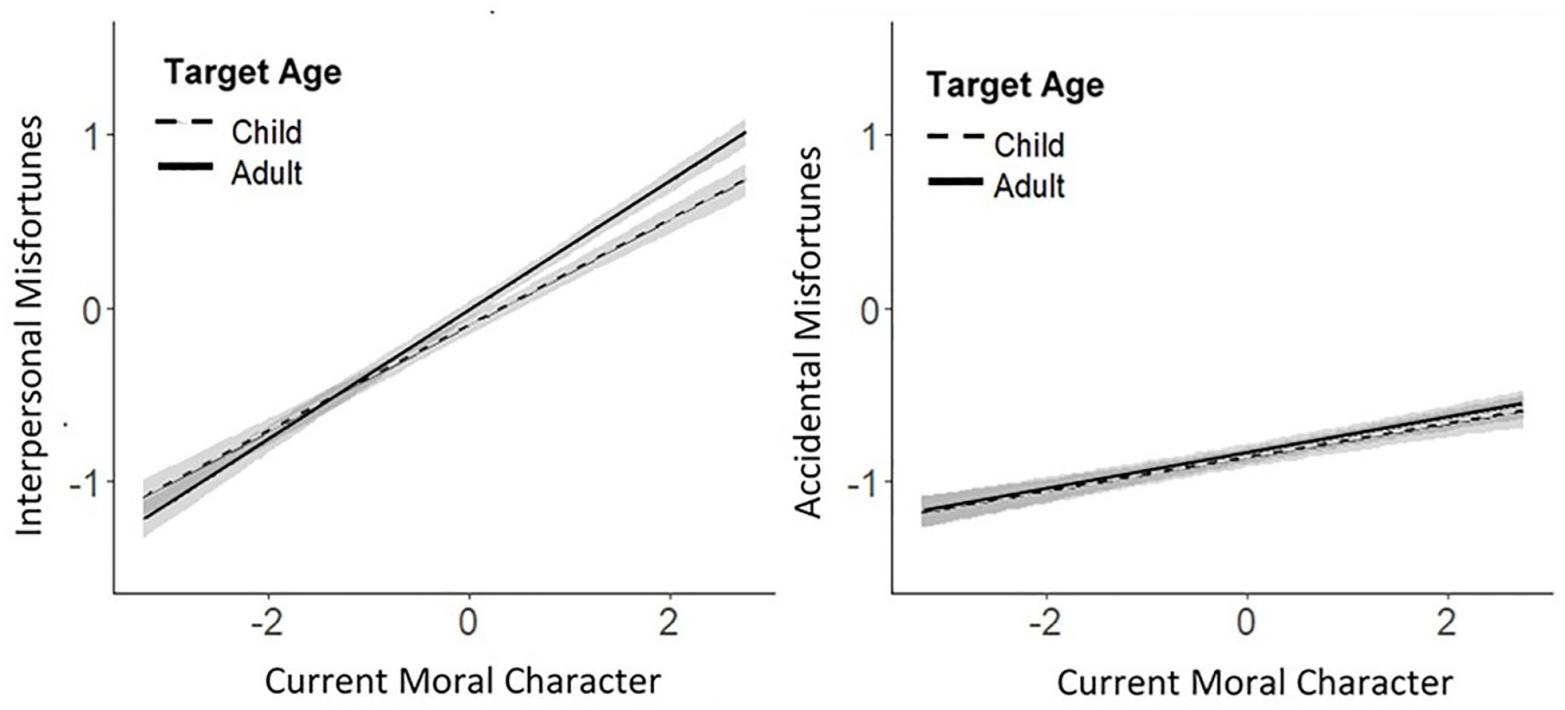
Regression, with 95% confidence bands, showing that the target’s age (adult or child) moderated the relationship between inferences about current moral character and forecasts about future interpersonal (but not accidental) misfortunes. Regression lines are plotted from data aggregated across all studies.

As hypothesized, the age of the transgressor (adult or child) moderated the strength of the relationship between inferences about current moral character and forecasts about future moral character, *b* = 0.055, 95% CI [0.030, 0.080], *p* < .001, although the relationship between current and future character was extremely strong regardless of whether evaluations were made about adults or children. The age of the transgressor (adult or child) also moderated the extent to which inferences about current moral character were associated with forecasts about future interpersonal misfortunes (*b* = 0.074, 95% CI [0.045, 0.10], *p <* .001), but not with forecasts about accidental misfortunes (*b* = 0.0006, 95% CI [-0.036, 0.037], *p* = .97). These effects are depicted in Figs 1 and 2.

**Fig 3:**
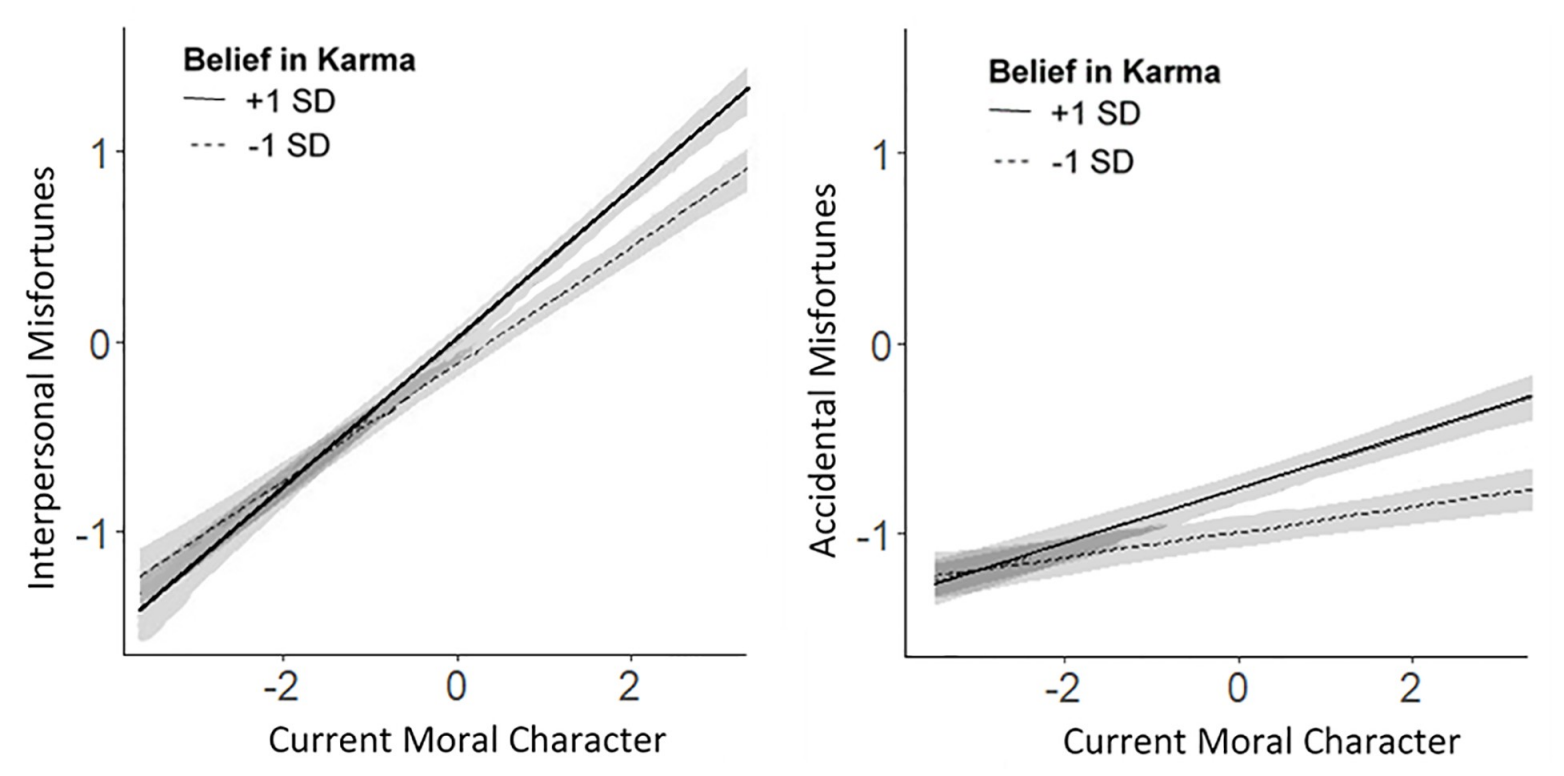
Regression, with 95% confidence bands, showing that participants’ belief in karma moderated the relations between inferences about current moral character and forecasts about future misfortunes. Regression lines are plotted from data aggregated across all studies.

Belief in karma also moderated the extent to which inferences about current moral character were associated with forecasts about future misfortunes, as predicted, for both interpersonal misfortunes (*b* = 0.054, 95% CI [0.006, 0.10], *p* = .029) and accidental misfortunes (*b* = 0.046, 95% CI [0.011, 0.081], *p* = .010). These effects are depicted in Fig 3. Belief in a just world also moderated the relation between inferences about current moral character and forecasts about interpersonal misfortunes (*b* = 0.044 [0.019, 0.069], *p* = < .001), but it did not moderate the relation with forecasts about accidental misfortunes (*b* = -0.003 [-0.020, 0.014], *p* = .72).

In constrast to our expectations (and past research), meta-analytic results provided no evidence that implicit personality theories—beliefs about the stability of moral character—moderated the relationship between inferences about current moral character and forecasts about future moral character, *b* = 0.004, 95% CI [-0.010, 0.017], *p* = .59.

## General discussion

How strongly do inferences about individuals’ current moral character predict forecasts about their future character and future misfortunes? Three studies tested which variables might moderate the strength of those relationships, by shaping assumptions about causality that determine the stability of character traits (and their consequences) across time. Results identified two moderating variables—transgressor age and belief in karma—that reveal how underlying assumptions about causality shape processes of character inferences and moral forecasts, whereas results revealed no evidence in support of implicit personality theories as a third hypothesized moderating effect.

It is notable that individual differences in implicit theories, the variable that seems to most directly assess the perceived stability/malleability of moral character traits, did not moderate any of the effects in any study. Although this hypothesis had not been directly tested in previous studies, it seems straightforward that people who generally believe in the stability of moral character would be more likely to use current moral character inferences to guide forecasts about future moral character. But none of the studies, nor the meta-analytic integration, provided any evidence that this was the case. We used the most appropriate individual difference measure that was available, i.e., a measure that assesses implicit theories specifically about *moral* character [29], which has previously been shown to be empirically distinct from implicit theories of other domains (e.g., intelligence), not reducible to demographic differences or response biases, and predictive of responses to moral transgressions [19, 33, 34]. Other, less-specific measures of implicit personality theories have also been shown to predict the likelihood of making dispositional inferences from observed behaviors [e.g., 20, 21]. Our results did not conceptually replicate those past findings, and thus contributes additional evidence (from high-powered preregistered studies) to the literature on how implicit theories may or may not influence social judgments, although future research is required to determine whether our null result is due to methodological factors or a more substantive reason.

Cross-cultural research supports the possibility that this null result may reflect a true non-effect: Compared to Americans, Koreans generally perceive lower levels of temporal stability in personality and—consistent with other work on implicit personality theories—are less inclined to infer dispositions from observed behaviors; but Koreans and Americans do *not* differ in expectations about the consistency between past moral behaviour and future moral behaviour [35]. Further research (using additional measures and methodologies) will be required to more comprehensively test whether perceivers’ beliefs about dispositional stability might possibly moderate the relationship between inferences and forecasts about moral character.

Results produced clear evidence of the moderating effects of belief in karma: People who more strongly believed in karma were more strongly inclined to use inferences about moral character to inform forecasts about future misfortunes. This finding conceptually replicates and extends research showing that belief in karma amplifies the perceived causal connection between past misdeeds and current misfortunes [23], by revealing an analogous effect in forecasts about the future: In addition to showing an increased willingness to attribute *specific* bad experiences to *specific* past bad deeds, this study confirms that belief in karma reflects a more general tendency to expect that one’s current character traits (determined by a collection of actions and dispositions) predict one’s likelihood of a variety of future experiences. A subset of these effects were unique to the belief in karma: Although belief in a just world also moderated the relation between moral inferences and forecasts about interpersonal misfortunes, only belief in karma moderated the relation between moral inferences and forecasts about accidental misfortunes. This result extends previous research, suggesting that—compared to secular justice beliefs—belief in karma may influence forecasts about a wide range of future outcomes. These findings also highlight how people’s understanding of karmic causality is deeply intertwined with how they understand moral character and how dispositions persist across time, not merely how they make consequentialist judgments about the just consequences of single actions. Despite the way that “karma” is sometimes used to explain a specific instance of retribution for a specific bad deed, our results show how the general moral character traits (produced by committing moral transgressions) are also an important part of these expectations.

The age of the transgressor was also a consistent moderator: The strong relation between inferences and forecasts about moral character was somewhat less strong when the transgressor was a child rather than an adult; similarly, the relation with forecasts about interpersonal (but not accidental) misfortunes was also somewhat less strong when the transgressor was a child. These results complement and extend previous research showing that different variables predict moral judgments about adults and about children [18]. Like several of those previously-documented differences, these moderating effects may reflect the perception that a child’s cognitive and behavioral capacities are still developing—with the consequence that inferences based on a child’s transgressions may provide somewhat less guidance for long-term moral forecasting. These inferences about children’s maturation we not directly captured in the available data (e.g., inferences about the transgressors’ agency and mental capabilities, described in the S1 File, do not directly tap into this construct), and future research may benefit from further investigating specific beliefs about the maturation and stability of children, and how these expectations shape inferences about children’s moral futures.

It was also notable child’s transgressions were only a little less informative for long-term moral forecasting: Although there were statistically significant moderating effects of the child/adult variable, the sizes of those moderating effects were small. Indeed, the very strong relation between current character inferences and forecasts about future moral character was present even when forecasting the futures of very young children, showing the generalizability of these character inference patterns across both adult and child targets. These results suggest that, although research on causal attribution suggests a “rocky road” from observations of individuals’ actions to inferences about their dispositions [15], there appears to be a much smoother highway from inferences about individuals’ current moral character to forecasts about their future moral character. When perceivers judge someone—even if that someone is a young child—to be a good or bad person, they very strongly expect that person to continue to be morally good or bad in the long-term future (and, somewhat less strongly, they expect them to experience appropriate karmic outcomes).

The results that we found were specifically from the *moral* domain, and it remains to be seen whether similar patterns would be found for other types of judgments. Judgments about morality may have uniquely potent implications for person perception and impression formation [4, 6, 36], and the perceived potential for dispositional change in the moral domain may be lower than it is for other traits and aptitudes, such as those pertaining to competence or self-control [25, 37]. If indeed the perceived capacity for dispositional change in the moral domain is low, it may have implications for expectations about redemption. Redemption—the idea that sinners might be saved, and that once-bad people can become better—is a common theme in literature and mythology, is a hallmark of inspiring life narratives, and is tacitly reflected in the belief that people are fundamentally good, despite their failings [25, 38, 39]. Although the vocabulary of redemption can be applied to many kinds of behavior change, the concept of redemption is most commonly understood to describe a change in moral character. To the extent that our results can be connected to this broader theme, the implication is that the expected likelihood of a wrong-doer’s redemption may vary in predictable ways (depending, for instance, on whether the wrong-doers is an adult or a child), and also that the expected likelihood of redemption is, in general, pretty low.

## Supporting information

S1 File (DOCX)
