# Supplementary material for: How strongly do moral character inferences predict forecasts of the future? Testing the moderating roles of transgressor age, implicit personality theories, and belief in karma
Source: PLoS One. 2020 Dec 21;15(12):e0244144. doi: 10.1371/journal.pone.0244144 (PMC7751861; doi:10.1371/journal.pone.0244144)
Supplement: S1 File — (DOCX) [file pone.0244144.s001.docx]

S1 File.

How strongly do moral character inferences predict forecasts of the future? Testing the moderating roles of transgressor age, implicit personality theories, and belief in karma

**Contents**

[Demographics across Studies 2](#_Toc55222476)

[Differences in Inferences and Forecasts across Experimental Conditions 3](#_Toc55222477)

[Moderators of Character Inferences Made from Perceived Wrongness of Actions 9](#_Toc55222478)

[Judgments about Mental Capabilities 11](#_Toc55222479)

[Future Character as a Predictor of Future Misfortunes 16](#_Toc55222480)

[Exploratory Moderators of the Association Between Character Inferences and Forecasts 18](#_Toc55222481)

[Internal Meta-Analysis 22](#_Toc55222482)

# Demographics across Studies

Table S1. Demographic composition of each sample

|  | Study 1 | Study 2 | Study 3A | Study 3B |
| --- | --- | --- | --- | --- |
| *N* | 299 | 660 | 309 | 218 |
| Gender |  |  |  |  |
| Female | 54% | 64% | 62% | 67% |
| Male | 46% | 36% | 38% | 33% |
| Age *M* (*SD*) | 36.89 (12.07) | 36.91 (12.29) | 36.22 (10.89) | 33.98 (11.36) |
| Ethnicity  Caucasian  Asian  Other or not provided | 76%  7%  17% | 79%  5%  16% | 79%  6%  15% | 76%  3%  21% |
| Median Income | $40,000 - $49,999 | $50,000 - $59,999 | $50,000 - $59,999 | $40,000 - $49,999 |
| Education  % with post-secondary degree | 70% | 75% | 66% | 66% |
| Religion |  |  |  |  |
| Christian | 48% | 51% | 50% | 51% |
| Non-religious, atheist, agnostic | 44% | 37% | 42% | 40% |
| Other | 8% | 12% | 8% | 9% |
| Parents | 42% | 47% | 46% | -- |

# Differences in Inferences and Forecasts across Experimental Conditions

To produce variability in character inferences and forecasts of the future, each study included an experimental manipulation that was expected to influence moral judgments. Study 1 manipulated whether transgressions were accidental or intentional, Study 2 manipulated whether actions were harmful or helpful, and Study 3 manipulated whether intentionally-harmful actions harm actually caused harm or were unharmful. All studies also manipulated whether actions were performed by an adult or a child. Below, we summarize how inferences and forecasts are affected by these manipulated variables.

Table 2 summarizes means across conditions, and mixed-effects models are used to test differences between conditions in Table 3 – 6. Due to correlations between the various inference and forecast measures (described in the main text), manipulated variables that produce harsher moral judgments also tend to lead to inferences of less mental capabilities, more negative current and future moral character, and a greater predicted likelihood of interpersonal misfortunes and accidental misfortunes, although these relationships were somewhat smaller for more distal forecasts compared to judgments of the action itself.

Table S2. Mean (SD) of evaluations, across all vignette conditions in each study.

|  |  | Wrongness of Action | | Current Character | | Future Character | | Interpersonal Misfortunes | | Accidental Misfortunes | |
| --- | --- | --- | --- | --- | --- | --- | --- | --- | --- | --- | --- |
| **Study 1** | |  |  |  |  |  |  |  |  |  |  |
| Adult | Intentional | 5.32 | (1.26) | 5.35 | (1.01) | 5.11 | (1.01) | 4.73 | (1.13) | 3.48 | (1.20) |
|  | Accidental | 1.67 | (0.98) | 2.70 | (1.03) | 2.69 | (0.99) | 3.52 | (1.25) | 3.14 | (1.24) |
| Child | Intentional | 4.58 | (1.48) | 4.68 | (1.13) | 4.27 | (1.25) | 4.34 | (1.13) | 3.34 | (1.20) |
|  | Accidental | 1.65 | (1.01) | 2.93 | (0.97) | 2.81 | (0.93) | 3.63 | (1.24) | 3.19 | (1.17) |
| **Study 2** | |  |  |  |  |  |  |  |  |  |  |
| Adult | Helpful | 1.78 | (1.07) | 1.99 | (0.77) | 2.16 | (0.89) | 3.69 | (1.19) | 3.54 | (1.04) |
|  | Neutral | 4.34 | (1.06) | 3.19 | (0.89) | 3.12 | (0.97) | 3.85 | (1.09) | 3.34 | (1.19) |
|  | Harmful | 8.04 | (1.32) | 5.24 | (1.03) | 4.94 | (1.13) | 4.81 | (1.02) | 3.55 | (1.09) |
| Child | Helpful | 1.65 | (1.07) | 2.23 | (0.89) | 2.33 | (0.90) | 3.84 | (1.19) | 3.55 | (1.06) |
|  | Neutral | 4.43 | (1.22) | 3.41 | (0.85) | 3.14 | (1.02) | 3.92 | (1.12) | 3.33 | (1.25) |
|  | Harmful | 7.14 | (1.48) | 4.38 | (1.06) | 4.08 | (1.17) | 4.24 | (1.11) | 3.48 | (1.15) |
| **Study 3a** | |  |  |  |  |  |  |  |  |  |  |
| Adult | Harmful | 5.28 | (1.22) | 5.47 | (0.92) | 5.19 | (1.13) | 4.59 | (1.11) | 3.40 | (1.14) |
|  | Non-harmful | 4.71 | (1.48) | 5.31 | (0.97) | 4.98 | (1.17) | 4.49 | (1.04) | 3.30 | (1.13) |
| Child | Harmful | 4.63 | (1.52) | 4.60 | (1.17) | 4.20 | (1.33) | 4.20 | (1.10) | 3.24 | (1.10) |
|  | Non-harmful | 3.95 | (1.65) | 4.44 | (1.13) | 4.01 | (1.28) | 4.02 | (1.17) | 3.21 | (1.14) |
| **Study 3b** | |  |  |  |  |  |  |  |  |  |  |
| Adult | Harmful | 5.28 | (1.33) | 5.44 | (0.94) | 5.04 | (1.18) | 4.55 | (1.18) | 3.44 | (1.26) |
|  | Non-harmful | 4.48 | (1.55) | 5.17 | (0.98) | 4.77 | (1.14) | 4.40 | (1.11) | 3.46 | (1.16) |
| Child | Harmful | 4.52 | (1.62) | 4.72 | (1.15) | 4.25 | (1.29) | 4.07 | (1.17) | 3.31 | (1.18) |
|  | Non-harmful | 4.08 | (1.60) | 4.56 | (1.08) | 4.14 | (1.20) | 4.15 | (1.26) | 3.32 | (1.32) |

*Note.*  Scores range from 1 – 7 (except for action wrongness in Study 2, where scores range from 1 – 9). Variables were scored with higher numbers indicating more negative judgments of the action, more negative character traits, and a greater likelihood of bad experiences. Models testing whether these scores are significantly different across conditions are available in the Supplemental Materials.

Table S3. Study 1: Effect of intentional/accidental and child/adult manipulations on all outcome measures.

|  | Wrongness | | Mental Capabilities^[[1]](#footnote-1)^ | | | Current Character | | | | | Future Character | | | | Interpersonal Misfortunes | | Accidental Misfortunes | |  |  |
| --- | --- | --- | --- | --- | --- | --- | --- | --- | --- | --- | --- | --- | --- | --- | --- | --- | --- | --- | --- | --- |
|  | *b [95% CI]* | *p* | | *b [95% CI]* | *p* | | *b [95% CI]* | | *p* | *b [95% CI]* | | | *p* | *b [95% CI]* | | *p* | *b [95% CI]* | *p* | |  |
| Intercept | 1.65 [1.50, 1.79] | <.001 | | 5.36 [5.23, 5.50] | <.001 | | 2.93 [2.81, 3.05] | <.001 | | 2.81 [2.69, 2.93] | | <.001 | | 3.63 [3.49, 3.76] | | <.001 | 3.19 [3.05, 3.32] | <.001 | | |
| Target Age | 0.03 [-0.16, 0.22] | .77 | | 0.75 [0.61, 0.90] | <.001 | | -0.23 [-0.40, -0.07] | | .006 | -0.12 [-0.29, 0.04] | | | .14 | -0.11 [-0.24, 0.02] | | .11 | -0.05 [-0.14, 0.04] | .30 | | |
| Intention | 2.93 [2.75, 3.12] | <.001 | | -0.61 [-0.74, -0.47] | <.001 | | 1.75 [1.58, 1.91] | <.001 | | 1.46 [1.30, 1.62] | | <.001 | | 0.71 [0.58, 0.84] | | <.001 | 0.15 [0.06, 0.25] | .001 | | |
| Age* Intention | 0.71 [0.45, 0.97] | <.001 | | -0.42 [-0.61, -0.23] | <.001 | | 0.90 [0.67, 1.14] | <.001 | | 0.96 [0.73, 1.19] | | <.001 | | 0.50 [0.32, 0.68] | | <.001 | 0.19 [0.06, 0.32] | .005 | | |

*Note.* Includes random intercepts and random effect of actor age. In all models, age was dummy coded: adult = 1 and child = 0. Intention was dummy coded: intentional harm = 1, accidental harm = 0.

Table S4. Study 2: Effect of harmful/helpful action and child/adult manipulations on all outcome measures.

|  | Wrongness | | Mental Capabilities | | | | Current Character | | | | | Future Character | | | | | Interpersonal Misfortunes | | Accidental  Misfortunes | |  |  |
| --- | --- | --- | --- | --- | --- | --- | --- | --- | --- | --- | --- | --- | --- | --- | --- | --- | --- | --- | --- | --- | --- | --- |
|  | *b [95% CI]* | *p* | | *b [95% CI]* | *p* | | | *b [95% CI]* | | *p* | *b [95% CI]* | | | *p* | | *b [95% CI]* | | *p* | *b [95% CI]* | *p* | |  |
| Intercept | 4.43 [4.26, 4.59] | <.001 | | 5.02 [4.87, 5.17] | <.001 | | | 3.41 [3.28, 3.53] | <.001 | | 3.14 [3.00, 3.28] | | <.001 | | | 3.92 [3.77, 4.07] | | <.001 | 3.33 [3.18, 3.49] | <.001 | | |
| Target Age | -0.08 [-0.28, 0.11] | .39 | | 0.64 [0.49, 0.79] | <.001 | | | -0.22 [-0.35, -0.08] | .002 | | -0.01 [-0.15, 0.12] | | .82 | | | -0.07 [-0.19, 0.06] | | .30 | 0.01 [-0.09, 0.10] | .84 | | |
| Harmful vs. Neutral | 2.71 [2.48, 2.94] | <.001 | | -0.37 [-0.59, -0.16] | | <.001 | | 0.97 [0.80, 1.15] | <.001 | | 0.95 [0.75, 1.14] | | <.001 | | | 0.32 [0.11, 0.54] | | .003 | 0.15 [-0.07, 0.36] | .18 | | |
| Helpful vs. Neutral | -2.77 [-3.00, -2.55] | <.001 | | 0.85 [0.64, 1.06] | <.001 | | | -1.18 [-1.35, -1.01] | <.001 | | -0.81 [-1.00, -0.62] | | | | <.001 | -0.07 [-0.29, 0.14] | | .49 | 0.22 [0.01, 0.43] | .043 | | |
| Age* Harmful | 0.99 [0.72, 1.25] | <.001 | | -0.26 [-0.47, -0.05] | .015 | | | 1.07 [0.88, 1.26] | <.001 | | 0.87 [0.68, 1.05] | | | | <.001 | 0.63 [0.46, 0.80] | | <.001 | 0.06 [-0.08, 0.19] | .42 | | |
| Age* Helpful | 0.21 [-0.05, 0.48] | .11 | | -0.40 [-0.61, -0.19] | <.001 | | | -0.02 [-0.21, 0.17] | .82 | | -0.16 [-0.34, 0.02] | | | | .089 | -0.09 [-0.26, 0.08] | | .33 | -0.02 [-0.15, 0.11] | .73 | | |

Note. Includes random intercepts and random effect of actor age.

Table S5. Study 3a: Effect of harmful/non-harmful outcome and child/adult manipulations on all outcome measures.

|  | Wrongness | | Mental Capabilities | | | | Current Character | | | | | Future Character | | | | | Interpersonal Misfortunes | | Accidental  Misfortunes | |  |  |
| --- | --- | --- | --- | --- | --- | --- | --- | --- | --- | --- | --- | --- | --- | --- | --- | --- | --- | --- | --- | --- | --- | --- |
|  | *b [95% CI]* | *p* | | *b [95% CI]* | *p* | | | *b [95% CI]* | | *p* | *b [95% CI]* | | | *p* | | *b [95% CI]* | | *p* | *b [95% CI]* | *p* | |  |
| Intercept | 3.95 [3.78, 4.12] | <.001 | | 4.92 [4.79, 5.05] | <.001 | | | 4.44 [4.32, 4.56] | <.001 | | 4.01 [3.87, 4.15] | | <.001 | | | 4.02 [3.90, 4.15] | | <.001 | 3.21 [3.08, 3.33] | <.001 | | |
| Target Age | 0.76 [0.55, 0.98] | <.001 | | 0.51 [0.36, 0.65] | <.001 | | | 0.87 [0.72, 1.02] | <.001 | | 0.97 [0.80, 1.13] | | <.001 | | | 0.47 [0.34, 0.60] | | <.001 | 0.09 [0.00, 0.17] | .039 | | |
| Harmful outcome | 0.68 [0.49, 0.88] | <.001 | | -0.13 [-0.24, -0.01] | | .031 | | 0.15 [0.02, 0.28] | .023 | | 0.19 [0.05, 0.33] | | .009 | | | 0.18 [0.07, 0.29] | | .002 | 0.04 [-0.05, 0.12] | .39 | | |
| Age* Outcome | -0.12 [-0.40, 0.16] | .39 | | 0.09 [-0.07, 0.25] | .26 | | | 0.01 [-0.18, 0.20] | .92 | | 0.02 [-0.18, 0.22] | | | | .84 | -0.08 [-0.24, 0.08] | | .31 | 0.06 [-0.05, 0.18] | .28 | | |

*Note.* Includes random intercepts and random effect of actor age. Outcome was dummy coded: harmful outcome = 1, nonharmful outcome = 0.

Table S6. Study 3b: Effect of harmful/non-harmful outcome and child/adult manipulations on all outcome measures.

|  | Wrongness | | Mental Capabilities | | | | Current Character | | | | | Future Character | | | | | Interpersonal Misfortunes | | Accidental  Misfortunes | |  |  |
| --- | --- | --- | --- | --- | --- | --- | --- | --- | --- | --- | --- | --- | --- | --- | --- | --- | --- | --- | --- | --- | --- | --- |
|  | *b [95% CI]* | *p* | | *b [95% CI]* | *p* | | | *b [95% CI]* | | *p* | *b [95% CI]* | | | *p* | | *b [95% CI]* | | *p* | *b [95% CI]* | *p* | |  |
| Intercept | 4.08 [3.87, 4.29] | <.001 | | 4.79 [4.63, 4.96] | <.001 | | | 4.56 [4.42, 4.71] | <.001 | | 4.14 [3.98, 4.31] | | <.001 | | | 4.15 [3.99, 4.31] | | <.001 | 3.32 [3.16, 3.49] | <.001 | | |
| Target Age | 0.41 [0.14, 0.68] | .003 | | 0.48 [0.31, 0.66] | <.001 | | | 0.61 [0.44, 0.78] | <.001 | | 0.63 [0.45, 0.81] | | <.001 | | | 0.25 [0.10, 0.40] | | <.001 | 0.13 [0.01, 0.25] | .030 | | |
| Harmful outcome | 0.44 [0.21, 0.67] | <.001 | | -0.17 [-0.31, -0.03] | | .022 | | 0.16 [0.00, 0.31] | .045 | | 0.10 [-0.04, 0.25] | | | | .17 | -0.08 [-0.21, 0.06] | | .25 | -0.01 [-0.13, 0.10] | .85 | | |
| Age* Outcome | 0.36 [0.04, 0.68] | .030 | | 0.02 [-0.18, 0.22] | .84 | | | 0.11 [-0.11, 0.33] | .33 | | 0.16 [-0.05, 0.37] | | | | .14 | 0.22 [0.03, 0.41] | | .025 | 0.00 [-0.16, 0.16] | .99 | | |

*Note.* Includes random intercepts and random effect of actor age.

# Moderators of Character Inferences Made from Perceived Wrongness of Actions

Table S7. Perceived wrongness, actor age, and their interaction, predicting current moral character.

|  | Study 1 | | |  | | | Study 2 | | |  | | | Study 3A | | | |  | | | Study 3B | | | | | |  |
| --- | --- | --- | --- | --- | --- | --- | --- | --- | --- | --- | --- | --- | --- | --- | --- | --- | --- | --- | --- | --- | --- | --- | --- | --- | --- | --- |
|  | |  | *b* [95% CI] | | *p* | | | | *b* [95% CI] | | | *p* | |  | | *b* [95% CI] | | | *p* | | |  | *b* [95% CI] | | *p* | |
| Intercept | |  | 0.01 [-0.07, 0.08] | | | .87 | | -0.01 [-0.07, 0.06] | | | .82 | | | | -0.31 [-0.39, -0.23] | | | <.001 | | | -0.23 [-0.32, -0.14] | | | <.001 | | |
| Wrongness | |  | 0.53 [0.49, 0.57] | | | <.001 | | 0.37 [0.35, 0.40] | | | <.001 | | | | 0.39 [0.34, 0.43] | | | <.001 | | | 0.38 [0.33, 0.43] | | | <.001 | | |
| Target Age | |  | -0.00 [-0.09, 0.09] | | | .99 | | -0.00 [-0.08, 0.07] | | | .93 | | | | 0.62 [0.53, 0.70] | | | <.001 | | | 0.46 [0.36, 0.56] | | | <.001 | | |
| Wrongness* Age | |  | 0.12 [0.08, 0.17] | | | <.001 | | 0.11 [0.08, 0.14] | | | <.001 | | | | -0.02 [-0.08, 0.04] | | | .55 | | | -0.02 [-0.08, 0.05] | | | .57 | | |

*Note.* Mixed-effect models including random intercepts and random effect of action wrongness nested within participants, to account for the within-subjects nature of the data. In all models, age was dummy coded with child = 0, adult = 1. Therefore, the main effect of “wrongness” represents the effect when evaluating child transgressors, “age” indicates the difference between evaluations of adults and children at the average level of wrongness, and the interaction indicates the change in the wrongness effect when evaluating an adult, rather than a child, transgressor.

Table S8. Perceived wrongness, implicit theories, and their interaction, predicting current moral character.

|  | Study 1 | | |  | | | Study 2 | | |  | | | Study 3A | | | |  | | |  |
| --- | --- | --- | --- | --- | --- | --- | --- | --- | --- | --- | --- | --- | --- | --- | --- | --- | --- | --- | --- | --- |
|  | |  | *b* [95% CI] | | *p* | | | | *b* [95% CI] | | | *p* | |  | | *b* [95% CI] | | | *p* |  |
| Intercept | |  | 0.02 [-0.04, 0.07] | | | .52 | | -0.00 [-0.05, 0.05] | | | .94 | | | | 0.00 [-0.06, 0.07] | | | .95 | |  |
| Wrongness | |  | 0.60 [0.57, 0.63] | | | <.001 | | 0.44 [0.42, 0.45] | | | <.001 | | | | 0.44 [0.40, 0.47] | | | <.001 | |  |
| Implicit theories | |  | -0.01 [-0.07, 0.04] | | | .60 | | 0.04 [-0.02, 0.09] | | | .17 | | | | 0.01 [-0.06, 0.07] | | | .85 | |  |
| Wrongness* Theories | |  | 0.01 [-0.02, 0.04] | | | .60 | | 0.01 [-0.01, 0.03] | | | .33 | | | | -0.02 [-0.05, 0.02] | | | .36 | |  |

*Note.* Mixed-effect models including random intercepts and random effect of action wrongness nested within participants, to account for the within-subjects nature of the data. In all models, age was dummy coded with child = 0, adult = 1.

# Judgments about Mental Capabilities

Across all studies, we had planned to investigate ratings of the transgressors’ mental capabilities as an alternative inference about transgressor’s character and a plausible explanation for why child transgressors are evaluated less negatively than adults who commit identical transgressions.

**Materials**

As part of the set of character inferences and predictions made about the targets of each vignette, participants completed 8 items that assessed judgments about the transgressor’s mental capabilities—including morally-relevant capabilities (“able to tell right from wrong,” “understanding how others are feeling,” “trying to do the right thing”), cognitive capabilities that are not explicitly relevant to moral behavior (“able to do things on purpose,” “self-restraint,” “intelligent thought”), and capabilities to feel (“pain,” “joy,” and “sadness”). These three subscales were analyzed separately, and as a composite measure of transgressors’ mental capabilities.

**Results and Discussion**

One potential explanation for why intentionally harmful children are evaluated less negatively than intentionally harmful adults (Study 1) is that children’s lack of agentic qualities makes the difference between “intentional” and “accidental” action less meaningful and thereby less blameworthy. Compared to adults, children were rated as possessing substantially less cognitive capabilities (Table 9), including capabilities relevant to morality (e.g., “able to tell right from wrong”) and capabilities relevant to non-moral action (e.g., “intelligent thought”). If this difference in perceived agency partially explains differences in moral evaluations of adults and children, then ratings of cognitive capabilities should moderate the intentionality effect in the same way that the adult/child difference moderates the intentionality effect. However, this data does not follow this pattern. Although there was a significant interaction between intentionality and ratings of (non-moral) cognitive capabilities in predicting wrongness judgments (Table 10), this effect was not consistent with the effect of actor age. As displayed in Figure 1, for intentional actions, children were evaluated more positively than adults (as described in the main text), but, for intentional actions, mental capability ratings were unassociated with wrongness ratings.

Likewise, ratings of targets’ mental capabilities also did not moderate the relationship between inferences about current character and future character (see Table 11), whereas the target’s age (adult vs. child) did moderate the relationship between inferences and forecasts (see Table 1 and Figure 1 in the main text).

Therefore, instead of reflecting a potential mediator of adult/child differences in evaluations, ratings of an actor’s mental capabilities are better interpreted as an outcome measure, similar to ratings of the target’s character traits. Table 12 – 14 display the correlations between wrongness judgments, moral character judgments, and mental capability attributions for adults and children across all studies. In Studies 1 and 3, there were moderately-sized correlations between ratings of action wrongness, badness of character, and lower capability to think and feel. This pattern is consistent with other studies documenting the dehumanized perceptions of immoral actors (Khamitov, Rotman, & Piazza, 2016; Stellar & Willer, 2018). These effects were of similar size for judgments of adults and children, consistent with our finding that the wrongness of actions is predictive of poor moral character when judging both children and adults. These findings were somewhat reduced in Study 2a, perhaps due to a lack of variability in the moral judgment stimuli: All actions were purposeful and potentially-harmful, and were rated as rather wrong by participants, therefore dehumanization effects may not be apparent within this more narrow range of evaluations.

Table S9. Mean (SD) level of mental capabilities attributed to adults and children across studies.

|  | Study 1 | | Study 2 | | Study 3A | |
| --- | --- | --- | --- | --- | --- | --- |
|  | Adult | Child | Adult | Child | Adult | Child |
| Mind (overall) | 5.61 (1.30) | 5.06 (1.19) | 5.61 (1.26) | 5.19 (1.16) | 5.41 (1.45) | 4.86 (1.19) |
| Non-moral agency | 5.68 (1.22) | 4.83 (1.32) | 5.71 (1.20) | 5.01 (1.30) | 5.58 (1.33) | 4.60 (1.39) |
| Moral Agency | 5.42 (1.56) | 4.58 (1.52) | 5.54 (1.49) | 4.82 (1.52) | 5.10 (1.76) | 4.20 (1.59) |
| Affective capabilities | 5.72 (1.39) | 5.78 (1.34) | 5.58 (1.37) | 5.75 (1.29) | 5.55 (1.53) | 5.77 (1.36) |

Table S10. Study 1: Mental capabilities and harmful intentions predicting wrongness judgments.

|  |  | *b* [95% CI] | *p* |  | *b* [95% CI] | *p* |
| --- | --- | --- | --- | --- | --- | --- |
| Intercept |  | 1.69  [1.59, 1.80] | <.001 |  | 1.70  [1.59, 1.81] | <.001 |
| Intention |  | 3.24  [3.11, 3.38] | <.001 |  | 3.19  [3.04, 3.34] | <.001 |
| Non-moral agency |  | -0.14  [-0.25, -0.03] | .043 |  |  |  |
| Non-moral agency*Intention |  | 0.17  [0.02, 0.31] | .035 |  |  |  |
| Moral agency |  |  |  |  | -0.10  [-0.23, 0.03] | .127 |
| Moral agency*Intention |  |  |  |  | 0.07  [-0.08, 0.23] | .344 |

*Figure S1*

*Mental capabilities predicting wrongness judgments (left) and transgressor age predicting wrongness judgments (right) for intentional and accidental actions.*

Table S11. Forecasts about future moral character predicted by (a) inferences about current moral character, (b) degree of mental capabilities attributed to the transgressor (composite of affective, moral, and non-moral agency), and (c) the interaction between current moral character and mind attribution.

|  | Study 1 | | Study 2 | | Study 3A | | Study 3B | |
| --- | --- | --- | --- | --- | --- | --- | --- | --- |
|  | *b*  [95% CI] | *p* | *b*  [95% CI] | *p* | *b*  [95% CI] | *p* | *b*  [95% CI] | *p* |
| Intercept | -0.00 [-0.04, 0.04] | .98 | -0.00 [-0.05, 0.05] | .99 | -0.02 [-0.09, 0.04] | .48 | -0.05 [-0.12, 0.03] | .24 |
| Mind | 0.02 [-0.01, 0.05] | .26 | -0.02 [-0.06, 0.01] | .18 | 0.08 [0.04, 0.12] | **<.001** | 0.05 [0.01, 0.09] | **.019** |
| Current Character | 0.87 [0.84, 0.90] | **<.001** | 0.81 [0.78, 0.84] | **<.001** | 0.90 [0.85, 0.95] | **<.001** | 0.85 [0.79, 0.91] | **<.001** |
| Mind * Character | 0.00 [-0.02, 0.02] | .91 | -0.00 [-0.02, 0.02] | .92 | -0.01 [-0.04, 0.02] | .61 | -0.04 [-0.08, -0.00] | **.043** |

Table S12. Study 1: Correlations between moral judgments and mental capability judgments, children (lower diagonal) and adults (upper diagonal).

|  | Wrongness | Character | Mind | Non-moral agency | Moral agency | Affective capabilities |
| --- | --- | --- | --- | --- | --- | --- |
| Wrongness |  | 0.83^***^ | -0.42^***^ | -0.36^***^ | -0.46^***^ | -0.34^***^ |
| Character | 0.75^***^ |  | -0.44^***^ | -0.37^***^ | -0.50^***^ | -0.36^***^ |
| Mind (overall) | -0.26^***^ | -0.38^***^ |  | 0.92^***^ | 0.95^***^ | 0.93^***^ |
| Non-moral agency | -0.11^**^ | -0.23^***^ | 0.89^***^ |  | 0.82^***^ | 0.78^***^ |
| Moral agency | -0.25^***^ | -0.39^***^ | 0.91^***^ | 0.81^***^ |  | 0.83^***^ |
| Affective capabilities | -0.31^***^ | -0.36^***^ | 0.76^***^ | 0.49^***^ | 0.50^***^ |  |

Table S13. Study 2: Correlations between moral judgments and mental capability judgments, children (lower diagonal) and adults (upper diagonal).

|  | Wrongness | Character | Mind | Non-moral agency | Moral agency | Affective capabilities |
| --- | --- | --- | --- | --- | --- | --- |
| Wrongness |  | 0.85^***^ | -0.37^***^ | -0.28^***^ | -0.47^***^ | -0.26^***^ |
| Character | 0.77^***^ |  | -0.46^***^ | -0.37^***^ | -0.55^***^ | -0.35^***^ |
| Mind (overall) | -0.48^***^ | -0.56^***^ |  | 0.93^***^ | 0.94^***^ | 0.92^***^ |
| Non-moral agency | -0.41^***^ | -0.45^***^ | 0.90^***^ |  | 0.83^***^ | 0.80^***^ |
| Moral agency | -0.54^***^ | -0.59^***^ | 0.91^***^ | 0.84^***^ |  | 0.77^***^ |
| Affective capabilities | -0.25^***^ | -0.36^***^ | 0.72^***^ | 0.44^***^ | 0.44^***^ |  |

Table S14. Study 3A: Correlations between moral judgments and mental capability judgments, children (lower diagonal) and adults (upper diagonal).

|  | Wrongness | Character | Mind | Non-moral agency | Moral agency | Affective capabilities |
| --- | --- | --- | --- | --- | --- | --- |
| Wrongness |  | 0.52^***^ | 0.03 | 0.05 | 0.03 | -0.01 |
| Character | 0.59^***^ |  | -0.09^*^ | -0.04 | -0.10^**^ | -0.11^**^ |
| Mind (overall) | 0.05 | -0.09^*^ |  | 0.93^***^ | 0.95^***^ | 0.93^***^ |
| Non-moral agency | 0.18^***^ | 0.09^*^ | 0.87^***^ |  | 0.84^***^ | 0.79^***^ |
| Moral agency | 0.11^**^ | 0.00 | 0.91^***^ | 0.81^***^ |  | 0.81^***^ |
| Affective capabilities | -0.17^***^ | -0.32^***^ | 0.68^***^ | 0.32^***^ | 0.40^***^ |  |

# Future Character as a Predictor of Future Misfortunes

In addition to the analyses regarding how current moral character inferences inform forecasts of the future, we had also intended to analyze future moral character as a predictor of future misfortunes, and to test whether forecasts of future character mediated the relationship between current character inferences and forecasts of future misfortunes. However, due to the very high correlation between inferences of current character and forecasts of future character, any mediation analysis would result in a high level of multicollinearity, and is therefore of dubious inferential utility.

Instead, we conducted a set of analyses that examined relations between forecasts of future moral character and forecasts of future misfortunes, and tested the effects of plausible moderating variables. Results of these analyses were similar when predicting future misfortunes from current character inferences (main text, Tables 3 and 4) or future character inferences (Table 15 and 16, below): Across all studies, future character inferences were associated with forecasts of future interpersonal misfortunes and (to a lesser extent) accidental misfortunes; transgressor’s age (adult or child) tended to moderate this relationship when forecasting interpersonal, but not accidental, misfortunes; and belief in karma tended to moderate this relationship for forecasts of both interpersonal and accidental misfortunes. This consistency is not surprising given the extremely high correlation between inferences of current moral character and future moral character, across all studies.

Table S15. Future character predicting future misfortunes, moderated by target age.

|  | Study 1 | | Study 2 | | Study 3A | | Study 3B | |
| --- | --- | --- | --- | --- | --- | --- | --- | --- |
|  | Interpersonal Misfortunes | | | | | | | |
|  | *b* [95% CI] | *p* | *b* [95% CI] | *p* | *b* [95% CI] | *p* | *b* [95% CI] | *p* |
| Intercept | 0.01  [-0.10, 0.11] | .92 | -0.01 [-0.09, 0.06] | .77 | -0.02 [-0.11, 0.07] | .71 | -0.02 [-0.14, 0.09] | .69 |
| Character | 0.42  [0.37, 0.47] | <.001 | 0.41 [0.36, 0.46] | <.001 | 0.39 [0.33, 0.45] | <.001 | 0.46 [0.38, 0.53] | <.001 |
| Target Age | -0.02  [-0.08, 0.04] | .58 | 0.02 [-0.05, 0.08] | .59 | 0.03 [-0.04, 0.11] | .42 | 0.05 [-0.04, 0.14] | .27 |
| Character*  Age | 0.06  [0.01, 0.11] | .013 | 0.05 [0.00, 0.10] | .047 | 0.04 [-0.02, 0.10] | .21 | -0.04 [-0.11, 0.04] | .35 |
|  | Accidental Misfortunes | | | | | | | |
|  | *b [95% CI]* | *p* | *b [95% CI]* | *p* | *b [95% CI]* | *p* | *b [95% CI]* | *p* |
| Intercept | 0.01  [-0.12, 0.13] | .90 | 0.01 [-0.08, 0.09] | .91 | -0.01 [-0.13, 0.10] | .82 | 0.01 [-0.12, 0.13] | .90 |
| Character | 0.14  [0.09, 0.18] | <.001 | 0.14 [0.10, 0.19] | <.001 | 0.11 [0.06, 0.15] | <.001 | 0.14 [0.09, 0.18] | <.001 |
| Target Age | -0.01  [-0.07, 0.04] | .63 | -0.01 [-0.06, 0.05] | .80 | 0.02 [-0.05, 0.08] | .60 | -0.01 [-0.07, 0.04] | .63 |
| Character*  Age | 0.01  [-0.03, 0.05] | .55 | -0.03 [-0.07, 0.01] | .12 | 0.01 [-0.04, 0.06] | .82 | 0.01 [-0.03, 0.05] | .55 |

*Note.* Mixed-effect models including random intercepts and random effect of character

Table S16. Future character predicting future misfortunes, moderated by target age.

|  | Study 1 | | Study 2 | | Study 3A | | Study 3B | |
| --- | --- | --- | --- | --- | --- | --- | --- | --- |
|  | Interpersonal Misfortunes | | | | | | | |
|  | *b* [95% CI] | *p* | *b* [95% CI] | *p* | *b* [95% CI] | *p* | *b* [95% CI] | *p* |
| Intercept | 0.00 [-0.10, 0.10] | .98 | 0.00 [-0.07, 0.07] | .93 | 0.01 [-0.08, 0.09] | .90 | 0.00 [-0.10, 0.11] | .96 |
| Character | 0.46 [0.42, 0.50] | <.001 | 0.44 [0.40, 0.48] | <.001 | 0.42 [0.37, 0.47] | <.001 | 0.46 [0.39, 0.52] | <.001 |
| Belief in Karma | 0.02 [-0.08, 0.12] | .75 | -0.01 [-0.08, 0.06] | .75 | 0.07 [-0.02, 0.15] | .12 | 0.20 [0.10, 0.30] | <.001 |
| Character*Karma | 0.04 [-0.00, 0.08] | .059 | 0.06 [0.02, 0.10] | .004 | 0.03 [-0.02, 0.08] | .18 | 0.12 [0.06, 0.19] | <.001 |
|  | Accidental Misfortunes | | | | | | | |
|  | *b [95% CI]* | *p* | *b [95% CI]* | *p* | *b [95% CI]* | *p* | *b [95% CI]* | *p* |
| Intercept | 0.00 [-0.12, 0.12] | .97 | 0.00 [-0.08, 0.08] | .96 | -0.00 [-0.12, 0.11] | .93 | 0.00 [-0.12, 0.12] | .97 |
| Character | 0.14 [0.11, 0.18] | <.001 | 0.12 [0.08, 0.15] | <.001 | 0.11 [0.08, 0.15] | <.001 | 0.14 [0.11, 0.18] | <.001 |
| Belief in Karma | 0.12 [-0.00, 0.24] | .058 | 0.04 [-0.04, 0.12] | .37 | 0.16 [0.05, 0.27] | .005 | 0.12 [-0.00, 0.24] | .058 |
| Character*Karma | 0.04 [0.01, 0.07] | .018 | 0.05 [0.01, 0.08] | .013 | 0.05 [0.01, 0.09] | .007 | 0.04 [0.01, 0.07] | .018 |

*Note.* Mixed-effect models including random intercepts and random effect of character

# Exploratory Moderators of the Association Between Character Inferences and Forecasts

To provide a comprehensive analysis of the data, we also performed exploratory analyses regarding whether individual differences in implicit theories of moral character and belief in a just world moderated the association between character inferences and forecasts of future misfortunes. There was no evidence that implicit theories moderated these associations (Tables 17 and 19), consistent with the general lack of explanatory power of this variable throughout these studies. There was some evidence that belief in a just world (Tables 18 and 20) moderated the association between character inferences and forecasts of interpersonal (but not accidental) misfortunes. This finding provides an interesting contrast to belief in karma, which moderated forecasts of accidental misfortunes as well as interpersonal outcomes. This provides further evidence of a divergence between secular justice beliefs, that are especially relevant in interpersonal contexts, and supernatural justice beliefs, which explain a broader variety of good and bad experiences that lack any human agency as the source of justice.

Table S17. Current character predicting future misfortunes, moderated by implicit theories.

|  | Study 1 | | Study 2 | | Study 3A | |
| --- | --- | --- | --- | --- | --- | --- |
| Interpersonal Misfortunes | | | | | | |
|  | *b* [95% CI] | *p* | *b* [95% CI] | *p* | *b* [95% CI] | *p* |
| Intercept | 0.00 [-0.10, 0.11] | .93 | -0.00 [-0.07, 0.07] | .99 | -0.01 [-0.10, 0.08] | .84 |
| Character | 0.39 [0.35, 0.43] | <.001 | 0.35 [0.31, 0.39] | <.001 | 0.39 [0.34, 0.45] | <.001 |
| Implicit Theories | -0.06 [-0.16, 0.05] | .28 | 0.01 [-0.06, 0.09] | .75 | -0.05 [-0.14, 0.04] | .29 |
| Character*  Theories | 0.01 [-0.02, 0.05] | .49 | 0.01 [-0.03, 0.05] | .55 | -0.02 [-0.07, 0.04] | .60 |
| Accidental Misfortunes | | | | | | |
|  | *b [95% CI]* | *P* | *b [95% CI]* | *p* | *b [95% CI]* | *p* |
| Intercept | 0.01 [-0.11, 0.13] | .89 | -0.00 [-0.09, 0.08] | .93 | -0.01 [-0.12, 0.11] | .90 |
| Character | 0.12 [0.09, 0.15] | <.001 | 0.07 [0.04, 0.11] | <.001 | 0.12 [0.08, 0.16] | <.001 |
| Implicit Theories | 0.00 [-0.12, 0.13] | .95 | -0.04 [-0.12, 0.05] | .40 | -0.01 [-0.13, 0.10] | .84 |
| Character*  Theories | 0.00 [-0.03, 0.03] | .99 | 0.03 [-0.00, 0.07] | .061 | -0.01 [-0.05, 0.03] | .62 |

*Note.* Mixed-effect models including random intercepts and random effect of character

Table S18. Current character predicting future misfortunes, moderated by belief in a just world.

|  |  | Study 1 | | |  | Study 2 | | |  | Study 3A | | |
| --- | --- | --- | --- | --- | --- | --- | --- | --- | --- | --- | --- | --- |
|  | Interpersonal Misfortunes | | | | | | | | | | | |
|  | *b* [95% CI] | | *SE* | *p* | *b* [95% CI] | | *SE* | *p* | *b* [95% CI] | | *SE* | *p* |
| Intercept | 0.01 [-0.10, 0.11] | | 0.05 | .92 | 0.00 [-0.07, 0.08] | | 0.04 | .93 | -0.01 [-0.10, 0.08] | | 0.05 | .82 |
| Character | 0.39 [0.35, 0.43] | | 0.02 | <.001 | 0.35 [0.31, 0.39] | | 0.02 | <.001 | 0.40 [0.34, 0.46] | | 0.03 | <.001 |
| Belief in a Just World | -0.10 [-0.20, 0.00] | | 0.05 | .060 | -0.11 [-0.18, -0.03] | | 0.04 | .004 | 0.05 [-0.04, 0.15] | | 0.05 | .24 |
| Character* BJW | 0.03 [-0.01, 0.06] | | 0.02 | .15 | 0.05 [0.01, 0.09] | | 0.02 | .009 | 0.06 [0.00, 0.11] | | 0.03 | .033 |
| Marginal R^2^/ Conditional R^2^ | .235 / .788 | | | | .208 / .728 | | | | .166 / .687 | | | |
|  | Accidental Misfortunes | | | | | | | | | | | |
|  | *b [95% CI]* | | *SE* | *p* | *b [95% CI]* | | *SE* | *p* | *b [95% CI]* | | *SE* | *p* |
| Intercept | 0.01 [-0.11, 0.13] | | 0.06 | .89 | 0.00 [-0.08, 0.08] | | 0.04 | .99 | -0.01 [-0.12, 0.11] | | 0.06 | .89 |
| Character | 0.12 [0.09, 0.15] | | 0.01 | <.001 | 0.08 [0.04, 0.11] | | 0.02 | <.001 | 0.12 [0.08, 0.16] | | 0.02 | <.001 |
| Belief in a Just World | -0.06 [-0.19, 0.06] | | 0.06 | .30 | 0.00 [-0.08, 0.08] | | 0.04 | .99 | -0.05 [-0.17, 0.06] | | 0.06 | .37 |
| Character* BJW | -0.01 [-0.03, 0.02] | | 0.01 | .72 | -0.00 [-0.04, 0.03] | | 0.02 | .91 | 0.02 [-0.02, 0.06] | | 0.02 | .38 |
| Marginal R^2^/ Conditional R^2^ | .025 / .836 | | | | .010 / .813 | | | | .017 / .820 | | | |

*Note.* Mixed-effect models including random intercepts and random effect of character

Table S19. Future character predicting future misfortunes, moderated by implicit theories.

|  | Study 1 | | Study 2 | | Study 3A | |
| --- | --- | --- | --- | --- | --- | --- |
| Interpersonal Misfortunes | | | | | | |
|  | *b* [95% CI] | *p* | *b* [95% CI] | *p* | *b* [95% CI] | *p* |
| Intercept | 0.00  [-0.10, 0.10] | .97 | -0.00 [-0.07, 0.07] | .99 | 0.01 [-0.08, 0.09] | .87 |
| Character | 0.46  [0.42, 0.50] | <.001 | 0.45 [0.41, 0.49] | <.001 | 0.42 [0.37, 0.47] | <.001 |
| Implicit Theories | -0.05  [-0.15, 0.05] | .31 | 0.01 [-0.06, 0.08] | .85 | -0.07 [-0.15, 0.02] | .11 |
| Character*  Theories | -0.00  [-0.04, 0.04] | .95 | 0.01 [-0.02, 0.05] | .47 | 0.01 [-0.04, 0.05] | .82 |
| Accidental Misfortunes | | | | | | |
|  | *b [95% CI]* | *p* | *b [95% CI]* | *p* | *b [95% CI]* | *p* |
| Intercept | 0.00  [-0.12, 0.12] | .96 | -0.00 [-0.08, 0.08] | .94 | -0.00 [-0.12, 0.11] | .96 |
| Character | 0.14  [0.11, 0.18] | <.001 | 0.12 [0.08, 0.16] | <.001 | 0.11 [0.08, 0.15] | <.001 |
| Implicit Theories | 0.01  [-0.11, 0.13] | .91 | -0.04 [-0.12, 0.04] | .33 | -0.02 [-0.13, 0.09] | .70 |
| Character*  Theories | -0.01  [-0.04, 0.02] | .53 | 0.03 [-0.01, 0.07] | .11 | -0.01 [-0.05, 0.02] | .57 |

*Note.* Mixed-effect models including random intercepts and random effect of character

Table S20. Future character predicting future misfortunes, moderated by belief in a just world.

|  | Study 1 | | Study 2 | | Study 3A | |
| --- | --- | --- | --- | --- | --- | --- |
| Interpersonal Misfortunes | | | | | | |
|  | *b* [95% CI] | *p* | *b* [95% CI] | *p* | *b* [95% CI] | *p* |
| Intercept | 0.00 [-0.10, 0.10] | .97 | 0.01 [-0.06, 0.07] | .86 | 0.00 [-0.08, 0.09] | .92 |
| Character | 0.46 [0.42, 0.50] | <.001 | 0.44 [0.40, 0.48] | <.001 | 0.42 [0.37, 0.47] | <.001 |
| Belief in a Just World | -0.09 [-0.19, 0.01] | .076 | -0.09 [-0.16, -0.02] | .010 | 0.03 [-0.05, 0.12] | .43 |
| Character*BJW | 0.03 [-0.01, 0.07] | .20 | 0.06 [0.02, 0.10] | .001 | 0.04 [-0.00, 0.09] | .056 |
| Accidental Misfortunes | | | | | | |
|  | *b [95% CI]* | *p* | *b [95% CI]* | *p* | *b [95% CI]* | *p* |
| Intercept | 0.00 [-0.12, 0.12] | .97 | 0.00 [-0.08, 0.08] | .99 | -0.00 [-0.12, 0.11] | .94 |
| Character | 0.14 [0.11, 0.18] | <.001 | 0.12 [0.08, 0.16] | <.001 | 0.11 [0.08, 0.15] | <.001 |
| Belief in a Just World | -0.06 [-0.18, 0.06] | .34 | 0.01 [-0.08, 0.09] | .90 | -0.06 [-0.17, 0.06] | .34 |
| Character*BJW | -0.01 [-0.04, 0.03] | .75 | 0.01 [-0.03, 0.05] | .60 | 0.01 [-0.02, 0.05] | .46 |

*Note.* Mixed-effect models including random intercepts and random effect of character

# Internal Meta-Analysis

Table S21. Meta-analytic estimates of the association between current character and future character, moderated by target age and implicit theories.

|  | *b* [95% CI] | *p* | *b* [95% CI] | *p* |
| --- | --- | --- | --- | --- |
| Character | 0.81  [0.78, 0.83] | <.001 | 0.86  [0.81, 0.90] | <.001 |
| Target Age | 0.17  [0.12, 0.22] | <.001 |  |  |
| Character*  Age | 0.06  [0.03, 0.08] | <.001 |  |  |
| Implicit theories |  |  | 0.03  [-0.02, 0.07] | .26 |
| Character* Theories |  |  | 0.004  [-0.01, 0.02] | .59 |

Table S22. Meta-analytic estimates of the association between current character and likelihood of future misfortunes, moderated by target age and belief in karma.

|  | Interpersonal Misfortunes | | | | | | |
| --- | --- | --- | --- | --- | --- | --- | --- |
|  | *b* [95% CI] | *p* | *b* [95% CI] | *p* | *b* [95% CI] | *p* |  |
| Character | 0.33  [0.29, 0.36] | <.001 | 0.38  [0.35, 0.41] | <.001 | 0.38  [0.35, 0.41] | <.001 |  |
| Target Age | 0.08  [0.05, 0.12] | <.001 |  |  |  |  |  |
| Character*  Age | 0.07  [0.04, 0.10] | <.001 |  |  |  |  |  |
| Belief in Karma |  |  | 0.07  [-0.003, 0.15] | .059 |  |  |  |
| Character*Karma |  |  | 0.05  [0.006, 0.10] | .029 |  |  |  |
| BJW |  |  |  |  | -0.06  [-0.16, 0.04] | .27 |  |
| Character*BJW |  |  |  |  | 0.04  [0.02, 0.07] | <.001 |  |
|  | Accidental Misfortunes | | | | | |  |
|  | *b* [95% CI] | *p* | *b* [95% CI] | *p* | *b* [95% CI] | *p* |  |
| Character | 0.10  [0.08, 0.12] | <.001 | 0.11  [0.08, 0.14] | <.001 | 0.11  [0.09, 0.13] | <.001 |  |
| Target Age | 0.01  [-0.02, 0.05] | .37 |  |  |  |  |  |
| Character*  Age | 0.001  [-0.04, 0.04] | .97 |  |  |  |  |  |
| Belief in Karma |  |  | 0.11  [0.04, 0.19] | .002 |  |  |  |
| Character*Karma |  |  | 0.05  [0.01, 0.08] | .010 |  |  |  |
| BJW |  |  |  |  | -0.03  [-0.08, 0.03] | .37 |  |
| Character*BJW |  |  |  |  | -0.003  [-0.02, 0.01] | .72 |  |

1. The association between intentional harm and reduced mental capabilities is consistent across items documenting morally-relevant mental capabilities (e.g., “is able to tell right from wrong,” *b* = -0.93 [-1.11, -0.75], *p* < .001), cognitive capabilities that are not explicitly moral (e.g., “able to plan,” *b* = -0.31 [-0.45, -0.17], *p* < .001), and emotional capabilities (e.g., “able to feel pain”, *b* = -0.58 [-0.72, -0.43], *p* < .001), consistent with past research regarding dehumanization of immoral actors. Studies 2a, 2b, and 3 showed similar patterns, therefore we report analyses combining all items into a single mental capability measure. [↑](#footnote-ref-1)
